# Supplementary material for: Realizing efficient blue and deep-blue delayed fluorescence materials with record-beating electroluminescence efficiencies of 43.4%
Source: Nat Commun. 2023 Apr 10;14:2019. doi: 10.1038/s41467-023-37687-3 (PMC10086064; doi:10.1038/s41467-023-37687-3)
Supplement: Supplementary file 1 — Supplementary Information [file 41467_2023_37687_MOESM1_ESM.pdf]

SUPPLEMENTARY INFORMATION

Realizing efficient blue and deep-blue delayed fluorescence materials with record-beating electroluminescence efficiencies of 43.4%

Yan Fu,[1] Hao Liu,[1] Ben Zhong Tang[2] and Zujin Zhao[1,*]

[1] State Key Laboratory of Luminescent Materials and Devices, Guangdong Provincial Key Laboratory of Luminescence from Molecular Aggregates, South China University of Technology, Guangzhou, 510640, China.
[2] School of Science and Engineering, Shenzhen Institute of Aggregate Science and Technology, The Chinese University of Hong Kong, Shenzhen, Guangdong 518172, China.
* Corresponding Author: Zujin Zhao (email address: mszjzhao@scut.edu.cn)

Synthesis and Characterization

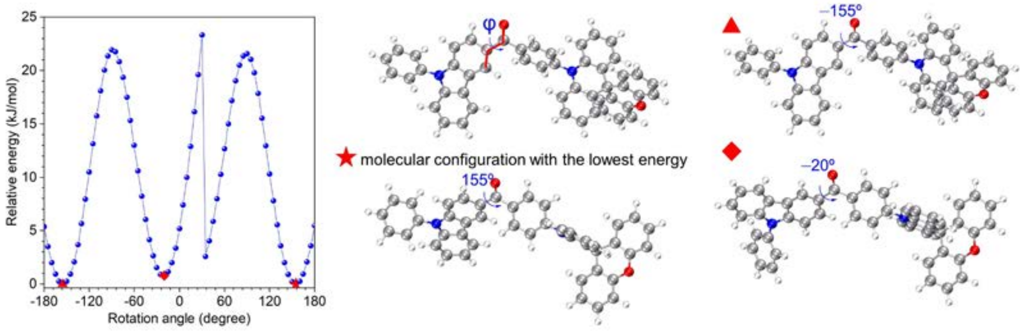

Supplementary Fig. 1 Conformational energy profile for the rotation of dihedral angle between carbazole and carbonyl of CBP-1. The configuration at the minimum point is represented by the red triangle, diamond, and pentagram, where the configuration represented by red pentagram is the one with the lowest energy.

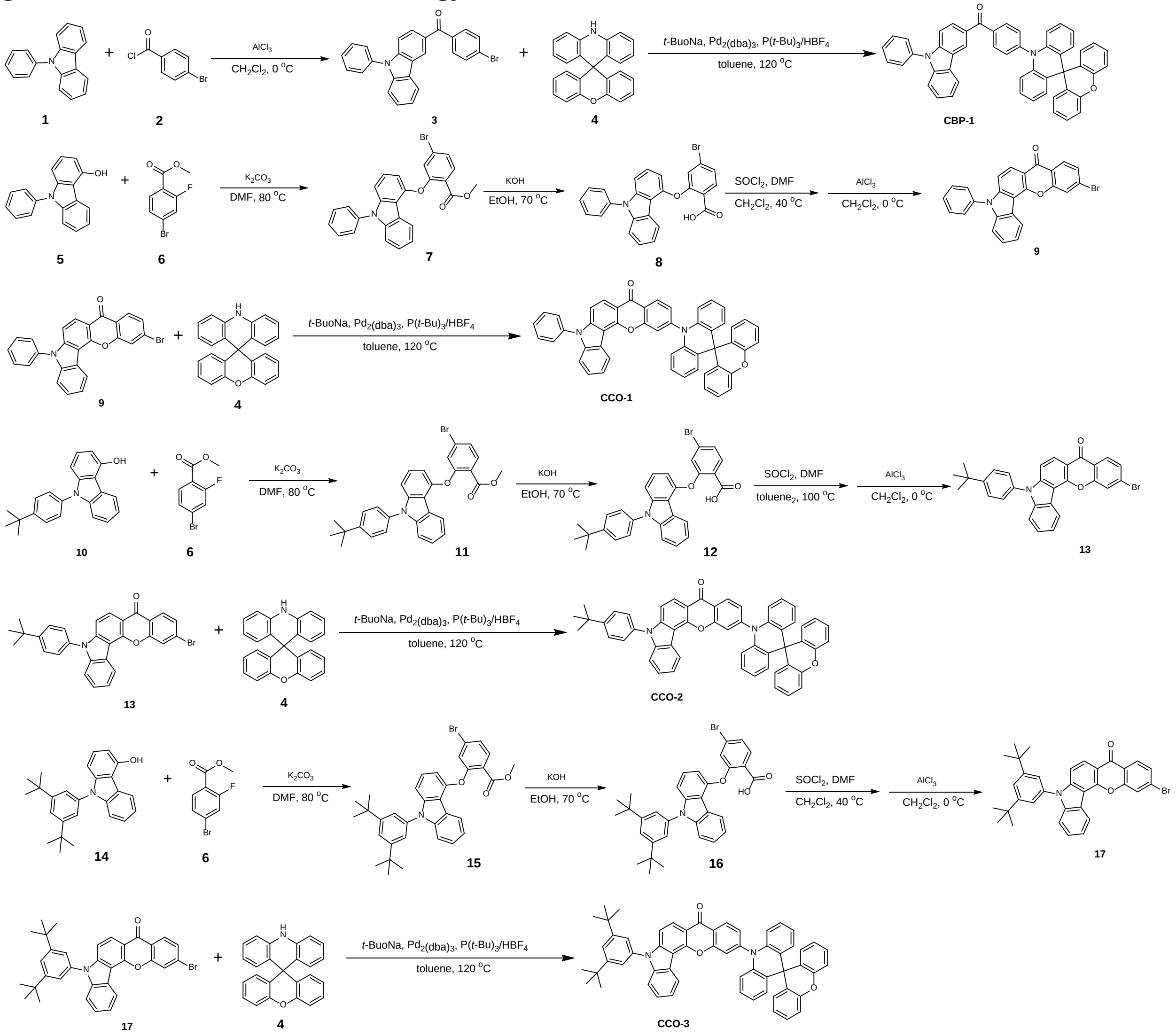

Supplementary Fig. 2 Synthetic routes of the new molecules. The synthesis methods and characterization data are described in detail below.

(4-Bromophenyl)(9-phenylcarbazol-3-yl)methanone (3): Aluminum trichloride (1.12 g, 8.40 mmol) was added to a stirred solution of 9-phenylcarbazole (**1**) (2.917 g, 12 mmol) and 4-bromobenzoyl chloride (**2**) (1.307 g, 6.0 mmol) in dehydrated dichloromethane (150 mL) in ice bath and stirred for 5 h. The reaction was quenched with ice water and hydrochloric acid (30 mL, 2.0 mol L^{-1}), and extracted with dichloromethane several times. The combined organic layers were washed twice with water, and then dried over anhydrous $MgSO_4$. After filtration and solvent evaporation under reduced pressure, the residue was purified by column chromatography on silica gel (dichloromethane/petroleum ether) to afford compound **3** as white solid in 61.7% yield (1.547 g). ^{1}H NMR (500 MHz, $CDCl_3$) δ 8.62–8.61 (m, 1H), 8.18–8.16 (m, 1H), 7.92–7.90 (m, 1H), 7.75–7.70 (m, 2H), 7.70–7.63 (m, 4H), 7.62–7.51 (m, 3H), 7.50–7.40 (m, 3H), 7.36–7.32 (m, 1H). ^{13}C NMR (125 MHz, $CDCl_3$) δ 195.39, 143.50, 141.79, 137.61, 136.85, 131.50, 130.10, 129.10, 128.48, 128.20, 127.14, 126.84, 126.69, 123.78, 123.30, 123.06, 120.98, 120.66, 110.31, 109.51. HRMS ($C_{25}H_{16}BrNO$): *m/z* 425.0413 [M^+, calcd 425.0415].

(4-(10H-Spiro[acridine-9,9'-xanthen]-10-yl)phenyl)(9-phenyl-9H-carbazol-3-yl)methanone (CBP-1): A mixture of compound **3** (1.50 g, 3.50 mmol), 10H-spiro[acridine-9,9'-xanthene] (**4**) (1.82 g, 5.25 mmol), sodium *tert*-butoxide (1.36 g, 14.00 mmol), tris(dibenzylideneacetone)dipalladium (0.129 g, 0.14 mmol) and tri-*tert*-butylphosphine tetrafluoroborate (0.164 g, 0.56 mmol) was added to a two-neck flask, evacuated and backfilled with dry nitrogen for three times. After that, 80 mL toluene was added into the flask. The reaction mixture was refluxed at 120 °C for 12 h and then cooled down to room temperature. The mixture was poured into water and extracted with dichloromethane several times. The combined organic layers were washed with water and dried over anhydrous $MgSO_4$. After filtration and solvent evaporation under reduced pressure, the residue was purified via silica-gel column chromatography using dichloromethane/petroleum ether as eluent. CBP-1 was obtained as blue-green solid in 82% yield (1.99 g). ^{1}H NMR (500 MHz, CD_2Cl_2) δ 8.81 (d, *J* = 1.4 Hz, 1H), 8.25 (d, *J* = 7.8 Hz, 1H), 8.19 (d, *J* = 8.2 Hz, 2H), 8.08–8.06 (m, 1H), 7.70–7.62 (m, 6H), 7.60–7.54 (m, 1H), 7.53–7.43 (m, 3H), 7.41–7.33 (m, 1H), 7.24–7.14 (m, 6H), 7.04–6.92 (m, 4H), 6.90–6.82 (m, 2H), 6.77–6.65 (m, 2H), 6.42 (d, *J* = 7.0 Hz, 2H). ^{13}C NMR (125 MHz, CD_2Cl_2) δ 195.58, 148.85, 144.49, 144.02, 142.31, 139.44, 139.32, 137.28, 133.18, 132.64, 132.20, 131.69, 130.54, 130.25, 129.67, 129.03, 128.61, 128.19, 127.72, 127.53, 127.28, 124.11, 124.02, 123.77, 123.54, 121.42, 121.36, 121.05, 116.43, 114.76, 110.76, 109.98, 45.08. HRMS ($C_{50}H_{32}N_2O_2$): *m/z* 692.2458 [M^+, calcd 692.2464].

Methyl 4-bromo-2-((9-phenyl-9H-carbazol-4-yl)oxy)benzoate (7): 9-Phenyl-9H-carbazol-4-ol (**5**) (3.90 g, 15 mmol) and potassium carbonate (4.14 g, 30 mmol) were added to a two-neck flask, evacuated and backfilled with dry nitrogen for three times. 25 mL anhydrous dimethylformamide (DMF) was added into the flask and heated to 60 °C. Then, anhydrous DMF solution (10 mL) of methyl 4-bromo-2-fluorobenzoate (**6**, 4.53 g, 19.5 mmol) was added to the reaction. After stirring at 80 °C for 12 h, the mixture was added into 40 mL of saturated NH_4Cl aqueous solution. Then, ethyl acetate was used to extract the aqueous phase. The organic phase was dried by rotary evaporation followed with column chromatography purification using dichloromethane/petroleum ether as eluent. Compound **7** was obtained as a colorless oily liquid in 75% yield (5.31 g). ^{1}H NMR (400 MHz, C_2D_6SO) δ 8.15 (d, *J* = 7.8 Hz, 1H), 7.89 (d, *J* = 8.4 Hz, 1H), 7.75–7.62 (m, 4H), 7.61–7.54 (m, 1H), 7.53–7.50 (m, 1H), 7.48–7.33 (m, 3H), 7.30–7.14 (m, 3H), 6.75 (d, *J* = 7.9 Hz, 1H), 3.74 (s, 3H). ^{13}C NMR (100 MHz, C_2D_6SO) δ 165.27, 156.54, 151.36, 142.72, 140.52, 137.07, 133.73, 130.68, 128.49, 127.63, 127.37, 127.19, 127.13, 126.73, 122.92, 122.07, 121.82, 121.20, 120.96, 114.35, 110.02, 109.26, 106.37, 55.35. HRMS ($C_{26}H_{18}BrNO_3$): *m/z* 471.0464 [M^+, calcd 471.0470].

4-Bromo-2-((9-phenyl-9H-carbazol-4-yl)oxy)benzoic acid (8): Compound **7** (3.00 g, 6.37 mmol) was dissolved in 40 mL of ethanol. Potassium hydroxide (3.57 g, 63.70 mmol) aqueous solution (20 mL) was added to the reaction. After stirring at 80 °C overnight, hydrochloric acid (2.0 mol L^{-1}) was used to neutralize the solution. The acquired solid was filtered and washed with water and dichloromethane. Compound **8** (2.62 g, 90%) was collected as a white solid after vacuum-drying. ^{1}H NMR (500 MHz, C_2D_6SO) δ 13.15 (s, 1H), 8.16 (d, *J* = 7.9 Hz, 1H), 7.88 (d, *J* = 8.4 Hz, 1H), 7.75–7.61 (m, 4H), 7.61–7.53 (m, 1H), 7.52–7.50 (m, 1H), 7.46–7.37 (m, 3H), 7.27–7.24 (m, 1H), 7.22–7.12 (m, 2H), 6.71 (d, *J* = 7.9 Hz, 1H). ^{13}C NMR (125 MHz, C_2D_6SO) δ 166.39, 156.27, 151.87, 142.68, 140.47, 137.09, 133.79, 130.72, 128.52, 127.69, 127.41, 127.29, 126.71, 126.50, 123.46, 123.07, 122.40, 121.31, 120.95, 114.17, 110.00, 108.93, 106.08. HRMS ($C_{25}H_{16}BrNO_3$): *m/z* 457.0308 [M^+, calcd 457.0314].

11-Bromo-5-phenylchromeno[3,2-c]carbazol-8(5H)-one (9): Compound **8** (2.50 g, 5.47 mmol) was added to a two-neck flask, evacuated and backfilled with dry nitrogen for three times. 20 mL dichloromethane was added to the flask, and then, thionyl chloride (0.98 g ,8.21 mmol) and DMF (1 mL) were added to the reaction. After stirring at 40 °C for 5 h, the organic phase was dried by rotary evaporation. Aluminum trichloride (1.12 g, 8.40 mmol) was added to a stirred solution of the organic phase in dehydrated dichloromethane (150 mL) in ice bath and stirred for 5 h. The reaction was quenched with ice water and hydrochloric acid (30 mL, 2.0 mol L^{-1}), and extracted with dichloromethane several times. The combined organic layers were washed twice with water, and then dried over anhydrous $MgSO_4$. After filtration and solvent evaporation under reduced pressure, the residue was purified by column chromatography on silica gel (dichloromethane/petroleum ether) to afford compound **9** as white solid in 75% yield (1.80 g).^{1}H NMR (500 MHz, CD_2Cl_2) δ 8.61–8.59 (m, 1H), 8.23–8.19 (m, 2H), 7.95 (d, *J* = 1.8 Hz, 1H), 7.72–7.64 (m, 2H), 7.62–7.48 (m, 5H), 7.48–7.42 (m, 2H), 7.34 (d, *J* = 8.8 Hz, 1H). ^{13}C NMR (125 MHz, CD_2Cl_2) δ 176.61, 157.29, 154.29, 146.34, 142.16, 137.72, 131.45, 129.83, 129.32, 129.22, 128.90, 128.57, 127.63, 125.26, 124.17, 122.92, 122.88, 122.69, 122.37, 116.11, 111.96, 111.72, 108.98. HRMS ($C_{25}H_{14}BrNO_2$): *m/z* 439.0202 [M^+, calcd 439.0208].

5-Phenyl-11-(10H-spiro[acridine-9,9'-xanthen]-10-yl)chromeno[3,2-c]carbazol-8(5H)-one (CCO-1): A mixture of compound **9** (1.54 g, 3.50 mmol), compound **4** (1.82 g, 5.25 mmol), sodium *tert*-butoxide (1.36 g, 14.00 mmol), tris(dibenzylideneacetone)dipalladium (0.129 g, 0.14 mmol) and tri-*tert*-butylphosphine tetrafluoroborate (0.164 g, 0.56 mmol) was added to a two-neck flask, evacuated and backfilled with dry nitrogen for three times. After that, 80 mL toluene was added into the flask. The reaction mixture was refluxed at 120 °C for 12 h and then cooled down to room temperature. The mixture was poured into water and extracted with dichloromethane several times. The combined organic layers were washed with water and dried over anhydrous $MgSO_4$. After filtration and solvent evaporation under reduced pressure, the residue was purified via silica-gel column chromatography using dichloromethane/petroleum ether as eluent. CCO-1 was obtained as blue-green solid in 85% yield (2.05 g). ^{1}H NMR (500 MHz, $CDCl_3$) δ 8.79 (d, *J* = 8.2 Hz, 1H), 8.68–8.66 (m, 1H), 8.43 (d, *J* = 8.8 Hz, 1H), 7.95 (d, *J* = 1.8 Hz, 1H), 7.70–7.67 (m, 2H), 7.64–7.55 (m, 4H), 7.54–7.49 (m, 1H), 7.49–7.44 (m, 2H), 7.43 (d, *J* = 8.7 Hz, 1H), 7.25–7.18 (m, 6H), 7.04–7.01 (m, 2H), 6.97–6.88 (m, 4H), 6.78–6.70 (m, 2H), 6.42–6.40 (m, 2H). ^{13}C NMR (125 MHz, $CDCl_3$) δ 175.93, 157.75, 153.41, 148.49, 146.45, 145.19, 140.88, 138.53, 136.46, 132.63, 131.97, 131.35, 130.20, 130.00, 129.85, 128.64, 127.71, 127.36, 127.28, 127.11, 126.40, 124.42, 123.72, 123.11, 121.78, 121.73, 121.46, 121.01, 116.04, 115.78, 115.11, 114.05, 110.82, 110.48, 107.90, 44.72. HRMS ($C_{50}H_{30}N_2O_3$): *m/z* 706.2251 [M^+, calcd 706.2256].

Methyl 4-bromo-2-((9-(4-(*tert*-butyl)phenyl)-9H-carbazol-4-yl)oxy)benzoate (11): 9-(4-(*tert*-butyl)phenyl)-9H-carbazol-4-ol (**10**) (4.73 g, 15 mmol) and potassium carbonate (4.14 g, 30 mmol) were added to a two-neck flask, evacuated and backfilled with dry nitrogen for three times. 25 mL anhydrous DMF was added into the flask and heated to 60 °C. Then, anhydrous DMF solution (10 mL) of compound **4** (4.53 g, 19.5 mmol) was added to the reaction. After stirring at 80 °C for 12 h, the mixture was added into 40 mL of saturated NH_4Cl aqueous solution. Then, ethyl acetate was used to extract the aqueous phase. The organic phase was dried by rotary evaporation followed with column chromatography purification using dichloromethane/petroleum ether as eluent. Compound **11** was obtained as a colorless oily liquid in 76% yield (6.01 g). ^{1}H NMR (500 MHz, CD_2Cl_2) δ 8.21–8.19 (m, 1H), 7.87 (d, *J* = 8.4 Hz, 1H), 7.69–7.62 (m, 2H), 7.56–7.48 (m, 2H), 7.43–7.37 (m, 2H), 7.36–7.33 (m, 2H), 7.27–7.19 (m, 2H), 7.17 (d, 1H), 6.72–6.71 (m, 1H), 3.81 (s, 3H), 1.44 (s, 9H). ^{13}C NMR (125 MHz, CD_2Cl_2) δ 166.82, 158.40, 152.59, 152.25, 144.40, 142.17, 135.93, 134.40, 128.55, 128.16, 127.87, 127.80, 127.57, 127.18, 124.16, 123.42, 122.71, 122.62, 121.53, 116.08, 110.90, 110.22, 107.38, 53.48, 36.03, 32.45. HRMS ($C_{30}H_{26}BrNO_3$): *m/z* 527.1090 [M^+, calcd 527.1096].

4-Bromo-2-((9-(4-(*tert*-butyl)phenyl)-9H-carbazol-4-yl)oxy)benzoic acid (12): Compound **11** (3.36 g, 6.37 mmol) was dissolved in 40 mL of ethanol. Potassium hydroxide (3.57 g, 63.70 mmol) aqueous solution (20 mL) was added to the reaction. After stirring at 80 °C overnight, hydrochloric acid (2.0 mol L^{-1}) was used to neutralize the solution. The acquired solid was filtered and washed with water and dichloromethane. Compound **12** (2.88 g, 88%) was collected as a white solid after vacuum-drying. ^{1}H NMR (500 MHz, C_2D_6SO) δ 13.15 (s, 1H), 8.15 (d, *J* = 7.9 Hz, 1H), 7.88 (d, *J* = 8.3 Hz, 1H), 7.75–7.63 (m, 2H), 7.60–7.56 (m, 2H), 7.52–7.50 (m, 1H), 7.46–7.28 (m, 3H), 7.29–7.21 (m, 1H), 7.21–7.04 (m, 2H), 6.70 (d, *J* = 7.9 Hz, 1H), 1.40 (s, 9H). ^{13}C NMR (125 MHz, C_2D_6SO) δ 166.39, 156.30, 151.85, 150.87, 142.72, 140.51, 134.47, 133.79, 127.63, 127.43, 127.27, 126.83, 126.66, 126.49, 123.45, 123.04, 122.38, 121.24, 120.84, 114.11, 110.07, 108.82, 106.16, 35.04, 31.63. HRMS ($C_{29}H_{24}BrNO_3$): *m/z* 513.0934 [M^+, calcd 513.0940].

11-Bromo-5-(4-(*tert*-butyl)phenyl)chromeno[3,2-c]carbazol-8(5H)-one (13): Compound **12** (2.80 g, 5.47 mmol) was added to a two-neck flask, evacuated and backfilled with dry nitrogen for three times. 20 mL toluene was added to the flask, and then, thionyl chloride (0.98 g, 8.21 mmol) and DMF (1 mL) were added to the reaction. After stirring at 100 °C for 48 h, the organic phase was dried by rotary evaporation. Aluminum trichloride (1.12 g, 8.40 mmol) was added to a stirred solution of the organic phase in dehydrated dichloromethane (150 mL) in ice bath and stirred for 5 h. The reaction was quenched with ice water and hydrochloric acid (30 mL, 2.0 mol L^{-1}), and extracted with dichloromethane several times. The combined organic layers were washed twice with water, and then dried over anhydrous $MgSO_4$. After filtration and solvent evaporation under reduced pressure, the residue was purified by column chromatography on silica gel (dichloromethane/petroleum ether) to afford compound **13** as white solid in 75% yield (2.03 g). ^{1}H NMR (500 MHz, CD_2Cl_2) δ 8.64–8.58 (m, 1H), 8.24–8.20 (m, 2H), 7.97 (d, *J* = 1.8 Hz, 1H), 7.71–7.64 (m, 2H), 7.56–7.54 (m, 1H), 7.54–7.48 (m, 3H), 7.48–7.41 (m, 2H), 7.36 (d, *J* = 8.7 Hz, 1H), 1.45 (s, 9H). ^{13}C NMR (125 MHz, CD_2Cl_2) δ 176.65, 157.32, 154.32, 153.08, 146.49, 142.29, 134.94, 129.30, 129.23, 128.89, 128.36, 127.96, 127.56, 125.17, 124.14, 122.84, 122.81, 122.72, 122.38, 116.02, 111.85, 111.83, 109.12, 36.11, 32.43. HRMS ($C_{29}H_{22}BrNO_2$): *m/z* 495.0828 [M^+, calcd 495.0834].

5-(4-(*tert*-Butyl)phenyl)-11-(10H-spiro[acridine-9,9'-xanthen]-10-yl)chromeno[3,2-c]carbazol-8(5H)-one (CCO-2): A mixture of compound **13** (1.73 g, 3.50 mmol), compound **4** (1.82 g, 5.25 mmol), sodium *tert*-butoxide (1.36 g, 14.00 mmol), tris(dibenzylideneacetone)dipalladium

(0.129 g, 0.14 mmol) and tri-*tert*-butylphosphine tetrafluoroborate (0.164 g, 0.56 mmol) was added to a two-neck flask, evacuated and backfilled with dry nitrogen for three times. After that, 80 mL toluene was added into the flask. The reaction mixture was refluxed at 120 °C for 12 h and then cooled down to room temperature. The mixture was poured into water and extracted with dichloromethane several times. The combined organic layers were washed with water and dried over anhydrous $MgSO_4$. After filtration and solvent evaporation under reduced pressure, the residue was purified via silica-gel column chromatography using dichloromethane/petroleum ether as eluent. CCO-2 was obtained as blue-green solid in 86% yield (2.30 g). ^{1}H NMR (400 MHz, $CDCl_3$) δ 8.79 (d, J = 8.3 Hz, 1H), 8.66 (d, J = 7.4 Hz, 1H), 8.42 (d, J = 8.8 Hz, 1H), 7.95 (d, J = 1.8 Hz, 1H), 7.68 (d, J = 8.5 Hz, 2H), 7.60–7.48 (m, 5H), 7.47–7.43 (m, 2H), 7.25–7.17 (m, 6H), 7.05–7.01 (m, 2H), 6.96–6.91 (m, 4H), 6.82–6.69 (m, 2H), 6.48–6.35 (m, 2H), 1.46 (s, 9H). ^{13}C NMR (125 MHz, CD_2Cl_2) δ 176.84, 159.15, 154.67, 153.14, 149.78, 147.53, 146.63, 142.39, 139.96, 134.97, 133.66, 133.14, 132.68, 131.18, 131.04, 130.26, 129.15, 128.46, 128.41, 128.00, 127.60, 125.36, 124.98, 124.22, 123.91, 122.94, 122.85, 122.57, 122.41, 117.38, 116.26, 115.60, 111.96, 111.89, 109.25, 45.99, 36.13, 32.44. HRMS ($C_{54}H_{38}N_2O_3$): *m/z* 762.2877 [M^+, calcd 762.2882].

Methyl 4-bromo-2-((9-(3,5-di-*tert*-butylphenyl)-9H-carbazol-4-yl)oxy)benzoate (15): 9-(3,5-Di-*tert*-butylphenyl)-9H-carbazol-4-ol (**14**) (5.57 g, 15 mmol) and potassium carbonate (4.14 g, 30 mmol) were added to a two-neck flask, evacuated and backfilled with dry nitrogen for three times. 25 mL anhydrous DMF) was added into the flask and heated to 60 °C. Then, anhydrous DMF solution (10 mL) of compound **6** (4.53 g, 19.5 mmol) was added to the reaction. After stirring at 80 °C for 12 h, the mixture was added into 40 mL of saturated NH_4Cl aqueous solution. Then, ethyl acetate was used to extract the aqueous phase. The organic phase was dried by rotary evaporation followed with column chromatography purification using dichloromethane/petroleum ether as eluent. Compound **15** was obtained as a colorless oily liquid in 76% yield (6.65 g). ^{1}H NMR (500 MHz, CD_2Cl_2) δ 8.21–8.19 (m, 1H), 7.87 (d, J = 8.4 Hz, 1H), 7.83–7.80 (m, 2H), 7.58–7.56 (m, 1H), 7.44 (d, J = 1.8 Hz, 2H), 7.39–7.37 (m, 2H), 7.35 (d, J = 1.8 Hz, 1H), 7.26–7.23 (m, 1H), 7.16 (d, J = 1.8 Hz, 1H), 6.73–6.72 (m, 1H), 3.82 (s, 3H), 1.41 (s, 18H). ^{13}C NMR (126 MHz, CD_2Cl_2) δ 166.83, 158.46, 152.57, 144.34, 142.11, 138.00, 134.43, 134.42, 129.04, 128.96, 128.90, 128.57, 127.85, 127.54, 124.19, 123.35, 122.95, 122.68, 121.48, 119.18, 116.07, 110.99, 107.51, 53.49, 36.38, 32.49. HRMS ($C_{34}H_{34}BrNO_3$): *m/z* 583.1716 [M^+, calcd 583.1722].

4-Bromo-2-((9-(3,5-di-*tert*-butylphenyl)-9H-carbazol-4-yl)oxy)benzoic acid (16): Compound **15** (3.71 g, 6.37 mmol) was dissolved in 40 mL of ethanol. Potassium hydroxide (3.57 g, 63.70 mmol) aqueous solution (20 mL) was added to the reaction. After stirring at 80 °C overnight, hydrochloric acid (2.0 mol L^{-1}) was used to neutralize the solution. The acquired solid was filtered and washed with water and dichloromethane. Compound **16** (3.12 g, 86%) was collected as a white solid after vacuum-drying. ^{1}H NMR (500 MHz, C_2D_6SO) δ 13.11 (s, 1H), 8.18–8.11 (m, 1H), 7.88 (d, J = 8.4 Hz, 1H), 7.58–7.57 (m, 1H), 7.51–7.49 (m, 1H), 7.46–7.36 (m, 5H), 7.30–7.10 (m, 3H), 6.72 (d, J = 7.9 Hz, 1H), 1.38 (s, 18H). ^{13}C NMR (125 MHz, C_2D_6SO) δ 166.41, 156.40, 152.97, 151.70, 142.65, 140.42, 136.56, 133.79, 127.73, 127.17, 126.78, 126.48, 123.33, 123.07, 122.11, 121.83, 121.25, 121.17, 120.83, 114.22, 110.05, 109.06, 106.25, 35.36, 31.63. HRMS ($C_{33}H_{32}BrNO_3$): *m/z* 569.1560 [M^+, calcd 569.1566].

11-Bromo-5-(3,5-di-*tert*-butylphenyl)chromeno[3,2-c]carbazol-8(5H)-one (17): Compound **16** (3.11 g, 5.47 mmol) was added to a two-neck flask, evacuated and backfilled with dry nitrogen for three times. 20 mL dichloromethane was added to the flask, and then, thionyl chloride (0.98 g, 8.21 mmol) and DMF (1 mL) were added to the reaction. After stirring at 40 °C for 5 h, The organic phase was dried by rotary evaporation. Aluminum trichloride (1.12 g, 8.40 mmol) was

added to a stirred solution of the organic phase in dehydrated dichloromethane (150 mL) in ice bath and stirred for 5 h. The reaction was quenched with ice water and hydrochloric acid (30 mL, 2.0 mol L^{-1}), and extracted with dichloromethane several times. The combined organic layers were washed twice with water, and then dried over anhydrous $MgSO_4$. After filtration and solvent evaporation under reduced pressure, the residue was purified by column chromatography on silica gel (dichloromethane/petroleum ether) to afford compound **17** as white solid in 76% yield (2.30 g). ^{1}H NMR (500 MHz, CD_2Cl_2) δ 8.63–8.61 (m, 1H), 8.25–8.20 (m, 2H), 7.96 (d, *J* = 1.7 Hz, 1H), 7.63–7.62 (m, 1H), 7.59–7.49 (m, 2H), 7.49–7.41 (m, 4H), 7.37 (d, *J* = 8.8 Hz, 1H), 1.41 (s, 18H). ^{13}C NMR (125 MHz, CD_2Cl_2) δ 176.62, 157.31, 154.41, 154.33, 146.44, 142.26, 137.06, 129.27, 129.22, 128.87, 127.59, 125.17, 124.14, 123.72, 122.81, 122.75, 122.72, 122.58, 122.37, 115.93, 111.92, 111.78, 109.23, 36.43, 32.47. HRMS ($C_{33}H_{30}BrNO_2$): *m/z* 551.1454 [M^+, calcd 551.1460].

5-(4-(*tert*-Butyl)phenyl)-11-(10H-spiro[acridine-9,9'-xanthen]-10-yl)chromeno[3,2-c]carbazol-8(5H)-one (CCO-3): A mixture of compound **17** (1.93 g, 3.50 mmol), compound **4** (1.82 g, 5.25 mmol), sodium *tert*-butoxide (1.36 g, 14.00 mmol), tris(dibenzylideneacetone)dipalladium (0.129 g, 0.14 mmol) and tri-*tert*-butylphosphine tetrafluoroborate (0.164 g, 0.56 mmol) was added to a two-neck flask, evacuated and backfilled with dry nitrogen for three times. After that, 80 mL toluene was added into the flask. The reaction mixture was refluxed at 120 °C for 12 h and then cooled down to room temperature. The mixture was poured into water and extracted with dichloromethane several times. The combined organic layers were washed with water and dried over anhydrous $MgSO_4$. After filtration and solvent evaporation under reduced pressure, the residue was purified via silica-gel column chromatography using dichloromethane/petroleum ether as eluent. CCO-3 was obtained as blue-green solid in 86% yield (2.46 g). ^{1}H NMR (500 MHz, $CDCl_3$) δ 8.79 (d, *J* = 8.2 Hz, 1H), 8.69–8.67 (m, 1H), 8.44 (d, *J* = 8.8 Hz, 1H), 7.96 (d, *J* = 1.9 Hz, 1H), 7.61–7.60 (m, 1H), 7.58–7.56 (m, 1H), 7.55–7.39 (m, 6H), 7.30–7.13 (m, 6H), 7.08–6.98 (m, 2H), 6.96–6.92 (m, 4H), 6.76–6.73 (m, 2H), 6.43–6.41 (m, 2H), 1.42 (s, 18H). ^{13}C NMR (125 MHz, $CDCl_3$) δ 175.91, 157.75, 153.44, 153.08, 148.49, 146.40, 145.29, 140.98, 138.55, 135.79, 132.63, 131.97, 131.36, 129.99, 129.85, 127.70, 127.24, 127.11, 126.32, 124.34, 123.72, 123.09, 122.57, 122.36, 121.68, 121.57, 121.45, 121.32, 121.00, 116.03, 114.92, 114.08, 110.66, 110.65, 108.10, 44.72, 35.23, 31.47. HRMS ($C_{58}H_{46}N_2O_3$): *m/z* 818.3502 [M^+, calcd 818.3508].

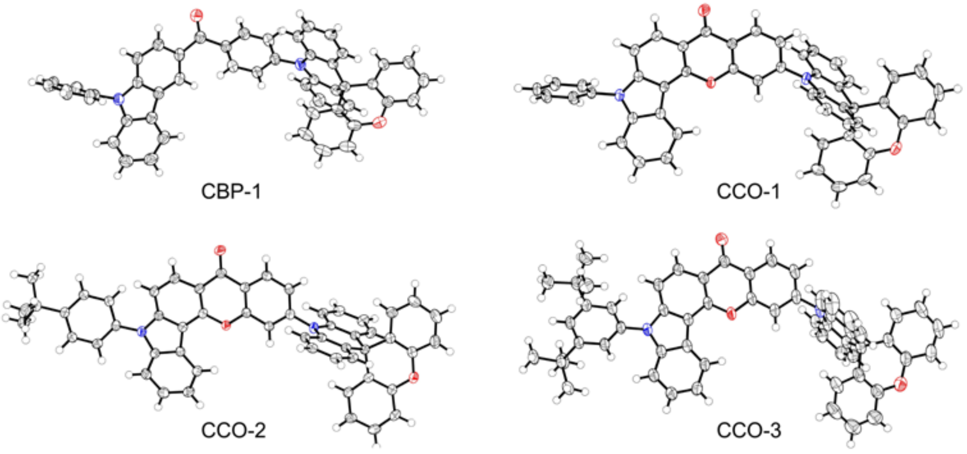

Supplementary Fig. 3 Crystal structure of CBP-1 (CCDC: 2209209), CCO-1 (CCDC: 2209210), CCO-2 (CCDC: 2209211), and CCO-3 (CCDC: 2209436). Thermal ellipsoids are drawn at the 50% probability level. Solvent molecules are omitted for clarity.

Crystal data for CBP-1 (CCDC: 2209209): $C_{50}H_{32}N_2O_2$, M_W = 692.24, monoclinic, *C*2/*c*, *a* = 32.3018(17), *b* = 9.2924(3), *c* = 27.0526(11) Å, α = 90°, β = 117.402(5)°, γ = 90°, *V* = 7209.1(6) Å^3, *Z* = 8, *D*c = 1.355 g cm^{-3}, μ = 1.305 mm^{-1}, *F*(000) = 3064, *T* = 150.00(10) K, R_1 (I > 2σ(I)) = 0.0591, wR_2 (I > 2σ(I)) = 0.1662, R_1 (all data) = 0.0693, wR_2 (all data) = 0.1759.
Crystal data for CCO-1 (CCDC: 2209210): $C_{50}H_{30}N_2O_3$, M_W = 706.23, triclinic, *P*-1, *a* = 9.01350(10), *b* = 14.2165(2), *c* = 15.3600(2) Å, α = 78.8470(10)°, β = 87.2790(10)°, γ = 88.4130(10)°, *V* = 1928.54(4) Å^3, *Z* = 2, *D*c = 1.423 g cm^{-3}, μ = 2.549 mm^{-1}, *F*(000) = 852, *T* = 149.99(10) K, R_1 (I > 2σ(I)) = 0.0532, wR_2 (I > 2σ(I)) = 0.1460, R_1 (all data) = 0.0598, wR_2 (all data) = 0.1516.
Crystal data for CCO-2 (CCDC: 2209211): $C_{54}H_{32}N_2O_3$, M_W = 762.29, triclinic, *P*-1, *a* = 8.9461(3), *b* = 15.3903(4), *c* = 19.0883(4) Å, α = 111.195(2) °, β = 95.198(2) °, γ = 101.159(2) °, *V* = 2366.70(12) Å^3, *Z* = 2, *D*c = 1.403 g cm^{-3}, μ = 3.679 mm^{-1}, *F*(000) = 1031, *T* = 150.00(10) K, R_1 (I > 2σ(I)) = 0.0507, wR_2 (I > 2σ(I)) = 0.1282, R_1 (all data) = 0.0620, wR_2 (all data) = 0.1349.
Crystal data for CCO-3 (CCDC: 2209436): $C_{58}H_{46}N_2O_3$, M_W = 818.97, triclinic, *P*-1, *a* = 13.1295(5), *b* = 14.4654(6), *c* = 14.8284(7) Å, α = 70.774(4) °, β = 66.590(4) °, γ = 71.614(4) °, *V* = 2384.3(2) Å^3, *Z* = 2, *D*c = 1.141 g cm^{-3}, μ = 0.545 mm^{-1}, *F*(000) = 864, *T* = 150.00(10) K, R_1 (I > 2σ(I)) = 0.0557, wR_2 (I > 2σ(I)) = 0.1575, R_1 (all data) = 0.0697, wR_2 (all data) = 0.1676.

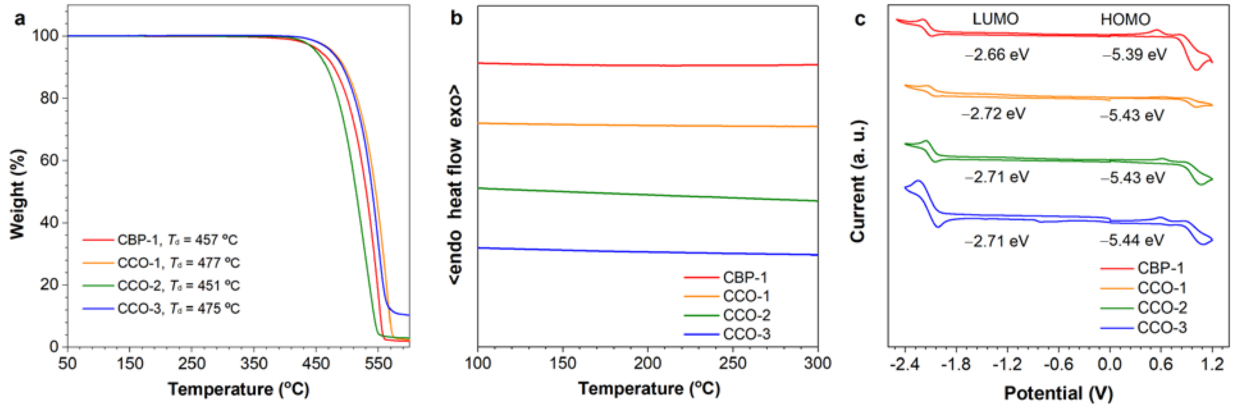

Supplementary Fig. 4 Thermal properties and electrochemical behaviors of CBP-1, CCO-1, CCO-2 and CCO-3. (**a**) TGA, (**b**) DSC thermograms and (**c**) electrochemical behaviors of these emitters. T_d is decomposition temperature, corresponding to 5% loss of initial weight. Cyclic voltammograms were measured in dichloromethane (HOMO energy level) and dimethylformamide (LUMO energy level) containing 0.1 M tetra-*n*-butylammonium hexafluorophosphate. Scanning rate: 50 mV s^{-1}.

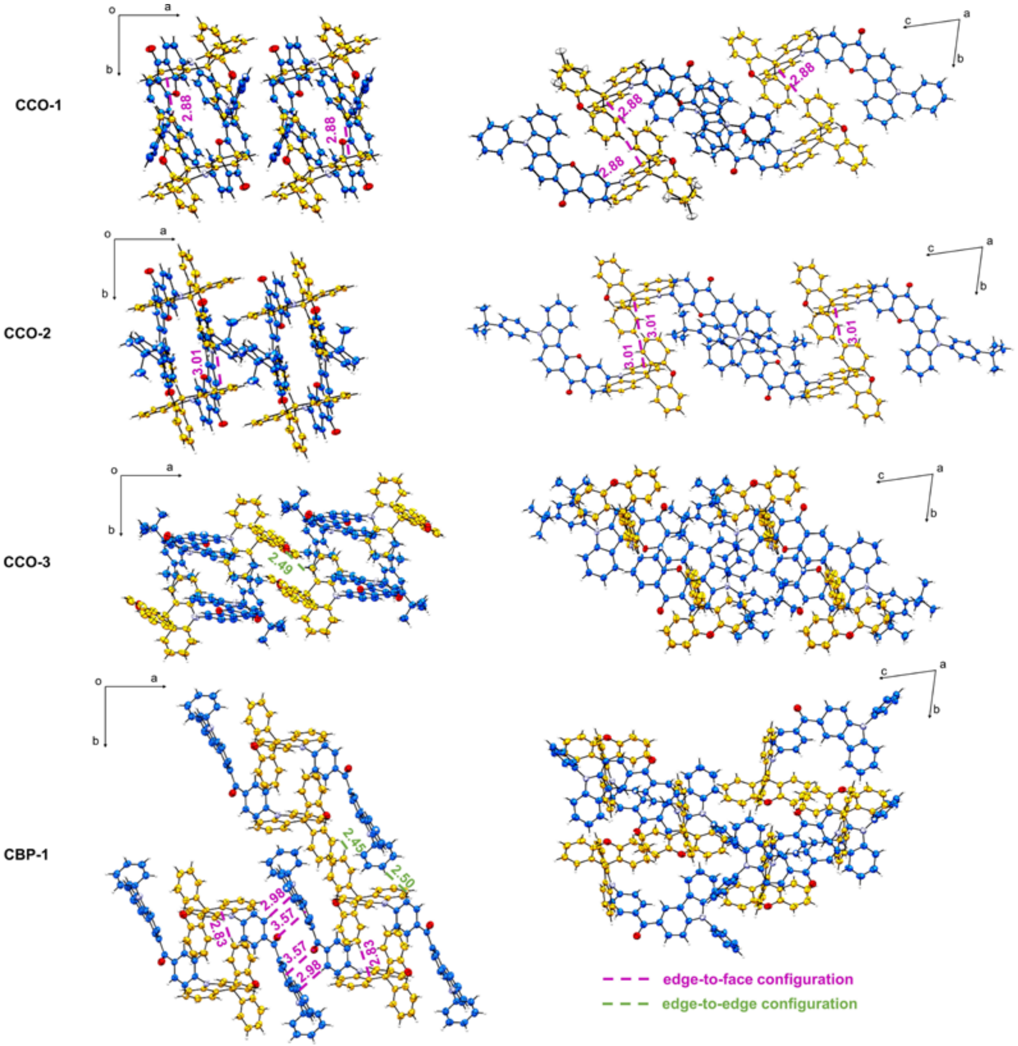

Supplementary Fig. 5 Packing arrangements of CCO-1, CCO-2, CCO-3 and CBP-1 in crystals. The purple dashed line represents edge-to-face configuration, and the green dashed line represents edge-to-edge configuration.

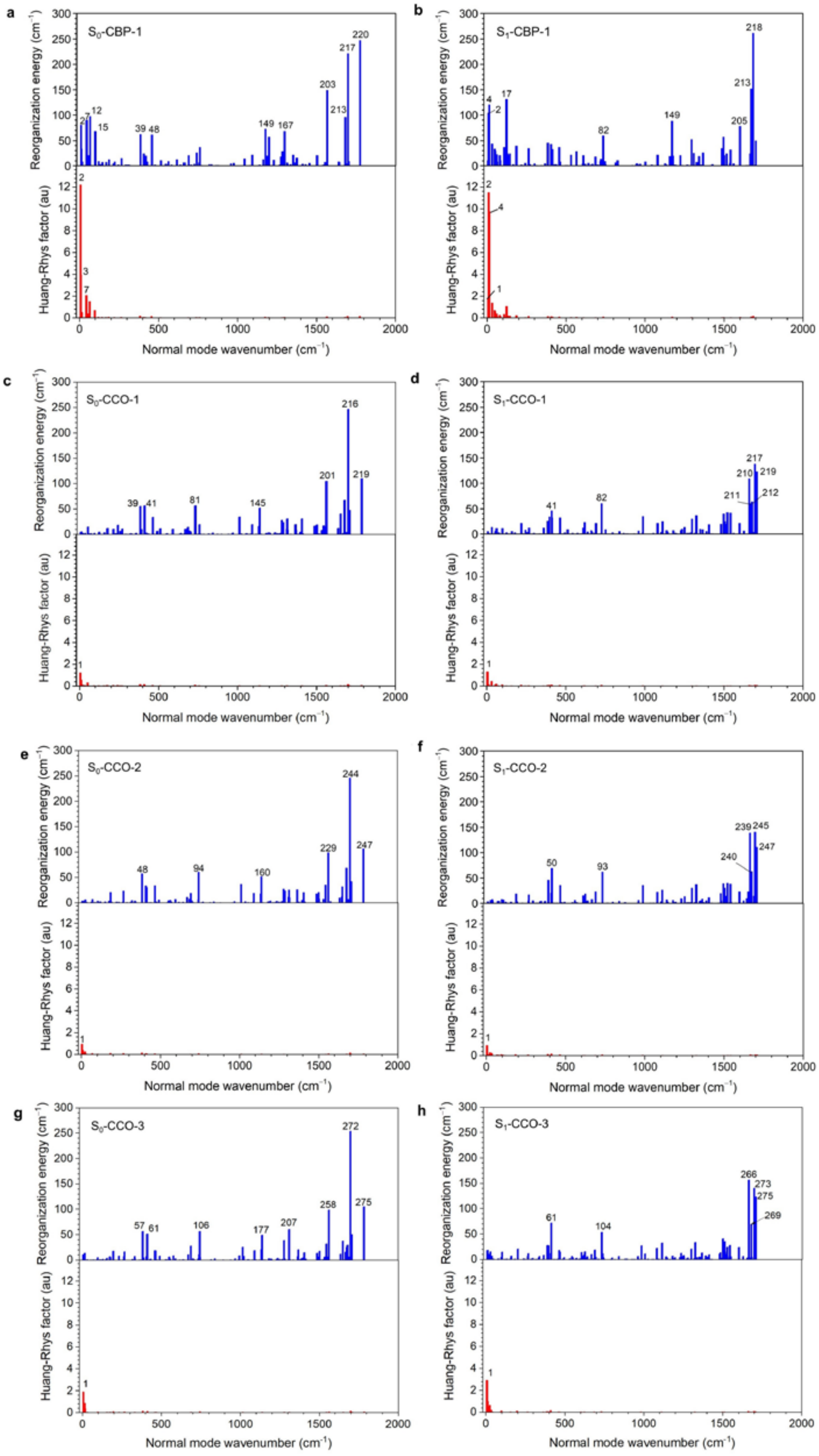

Supplementary Fig. 6 Decomposition of *λ* and *S*. Plots of calculated *λ* and *S* of S_0 and S_1 states vs. the normal modes of frequency for (**a, b**) CBP-1, (**c, d**) CCO-1, (**e, f**) CCO-2 and (**g, h**) CCO-3.

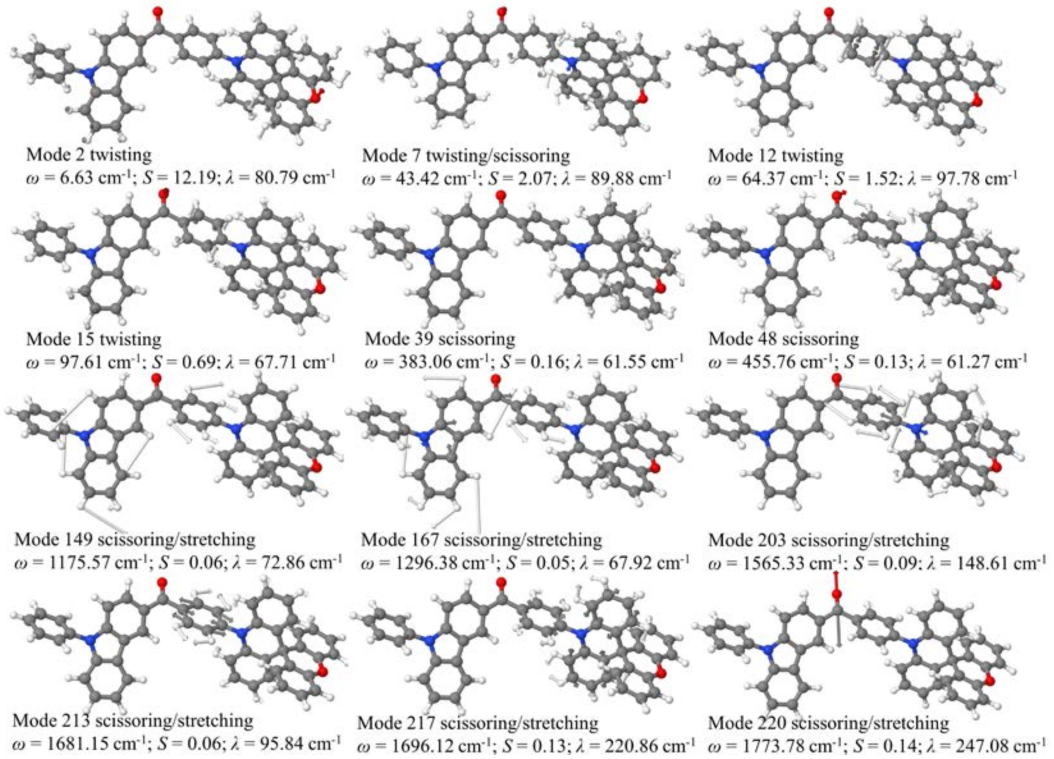

Supplementary Fig. 7 The dominant vibration mode for the displacements of the nuclear coordinates of S_0 state for CBP-1. The vibration frequency, S and λ for each vibration mode are indicated.

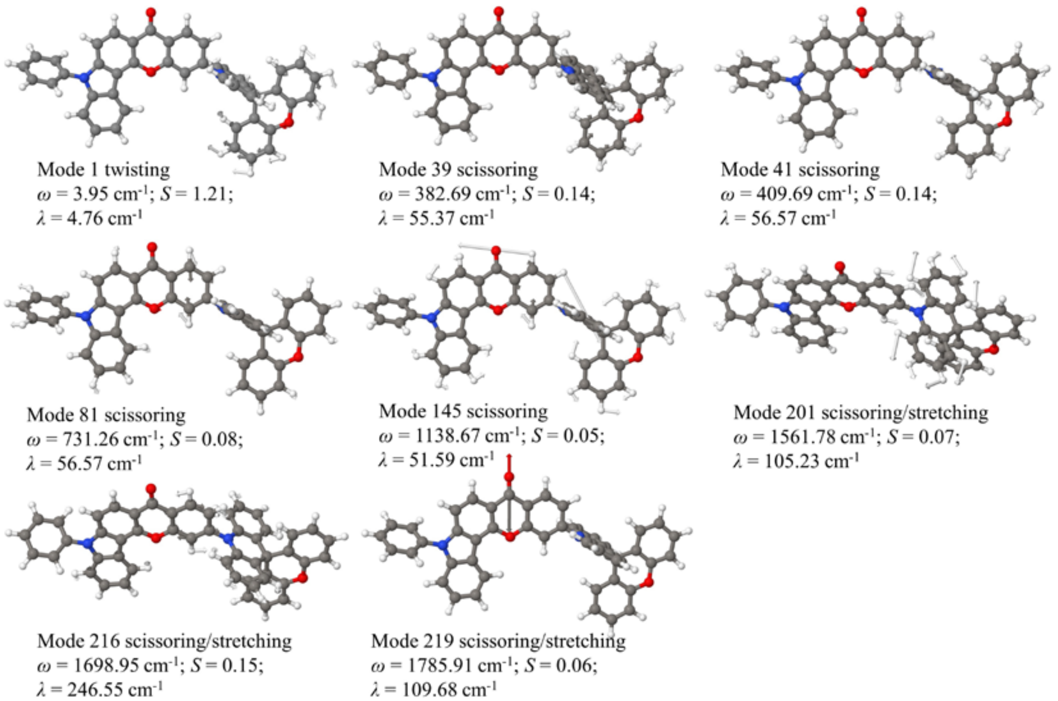

Supplementary Fig. 8 The dominant vibration mode for the displacements of the nuclear coordinates of S_0 state for CCO-1. The vibration frequency, S and λ for each vibration mode are indicated.

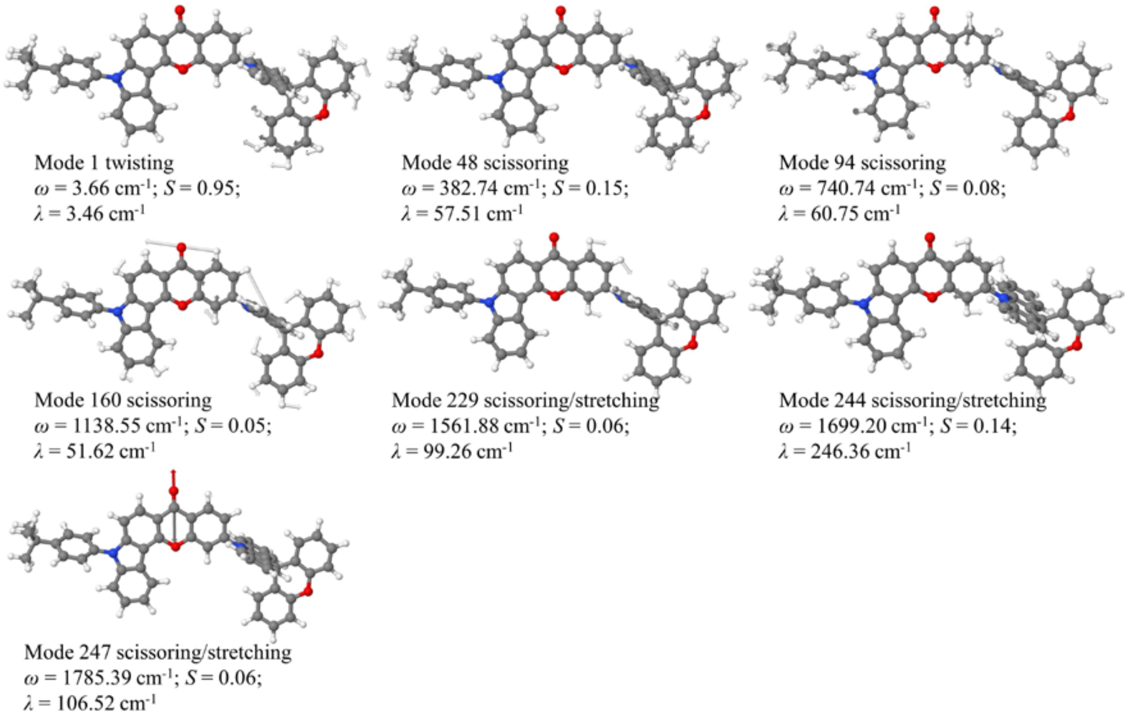

Supplementary Fig. 9 The dominant vibration mode for the displacements of the nuclear coordinates of S_0 state for CCO-2. The vibration frequency, S and λ for each vibration mode are indicated.

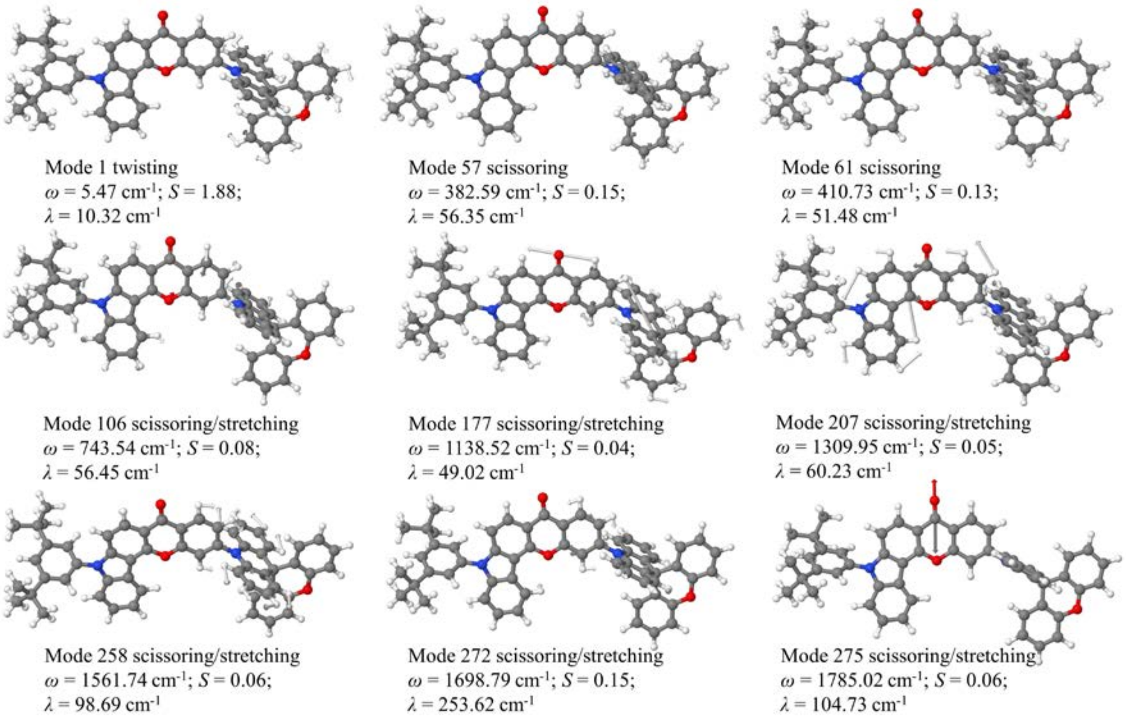

Supplementary Fig. 10 The dominant vibration mode for the displacements of the nuclear coordinates of S_0 state for CCO-3. The vibration frequency, S and λ for each vibration mode are indicated.

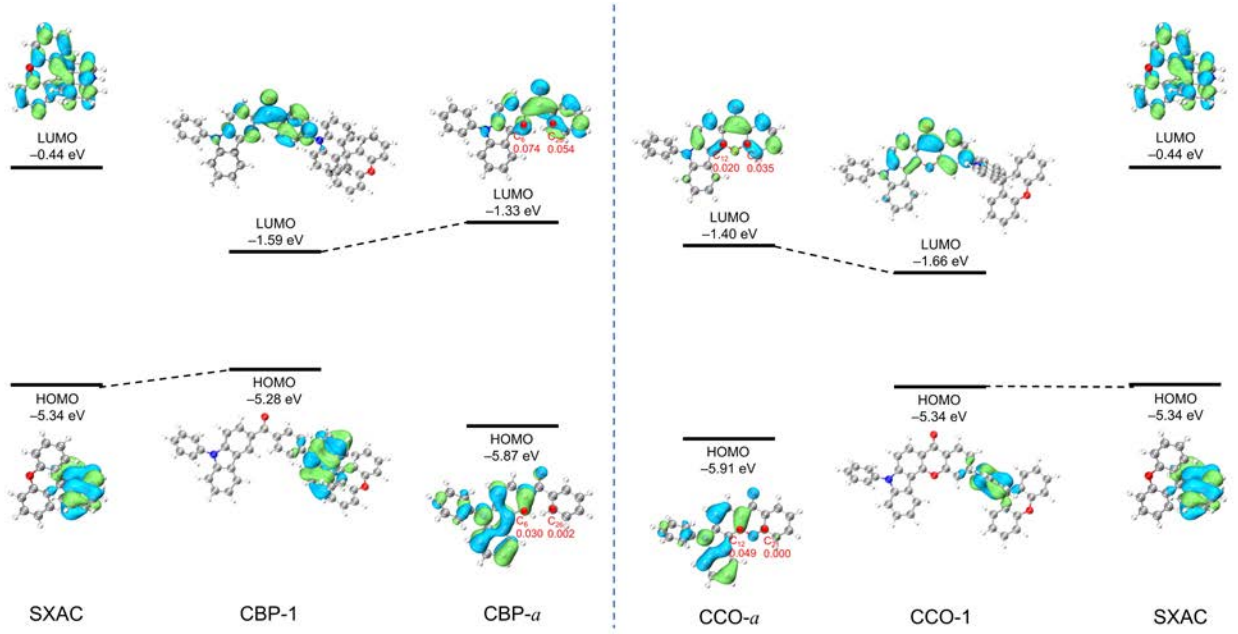

Supplementary Fig. 11 The energy levels and orbital distributions of CBP-1, CCO-1, CBP-*α*, CCO-*α* and SXAC. The red data represent the orbital coefficients at different positions.

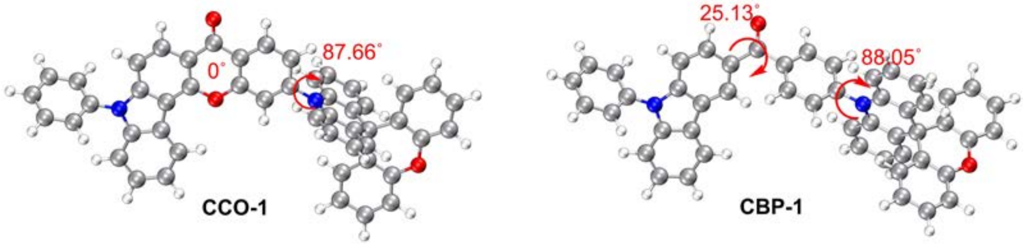

Supplementary Fig. 12 Calculated dihedral angles in CBP-1 and CCO-1. The optimized structures of CBP-1 and CCO-1 in S_0 state.

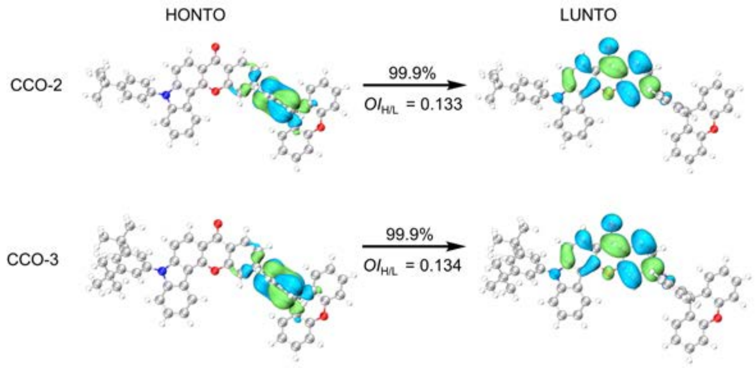

Supplementary Fig. 13 The NTO analysis of S_1 states for CCO-2 and CCO-3. The overlap integrals between HONTO and LUNTO are indicated.

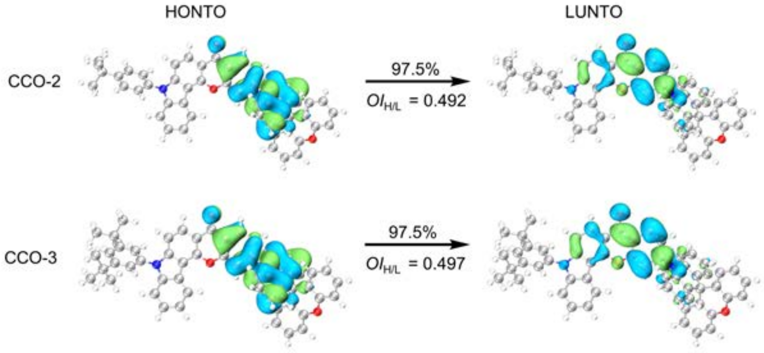

Supplementary Fig. 14 The NTO analysis of T_1 states for CBP-1, CCO-1, CCO-2 and CCO-3. The overlap integrals between HONTO and LUNTO are indicated.

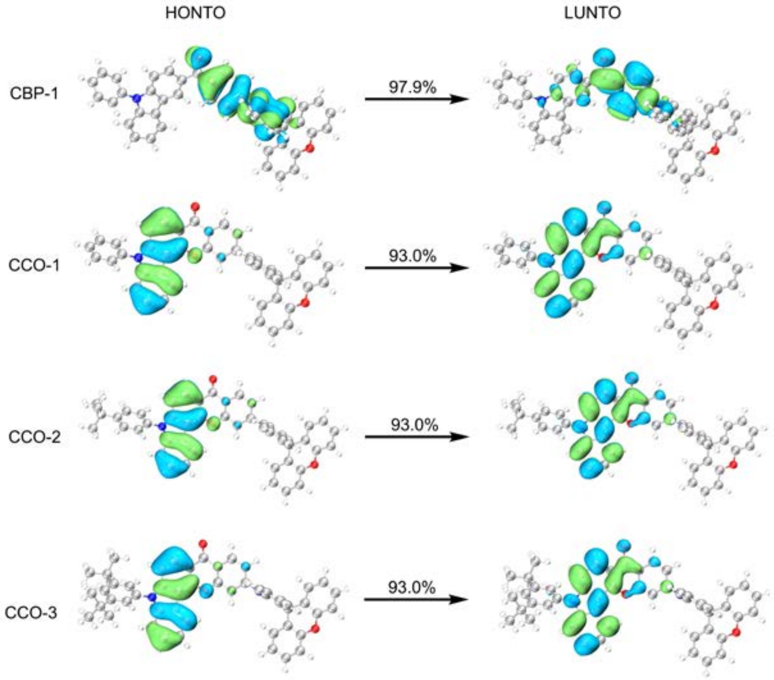

Supplementary Fig. 15 The NTO analysis of T_2 states for CBP-1, CCO-1, CCO-2 and CCO-3. The overlap integrals between HONTO and LUNTO are indicated.

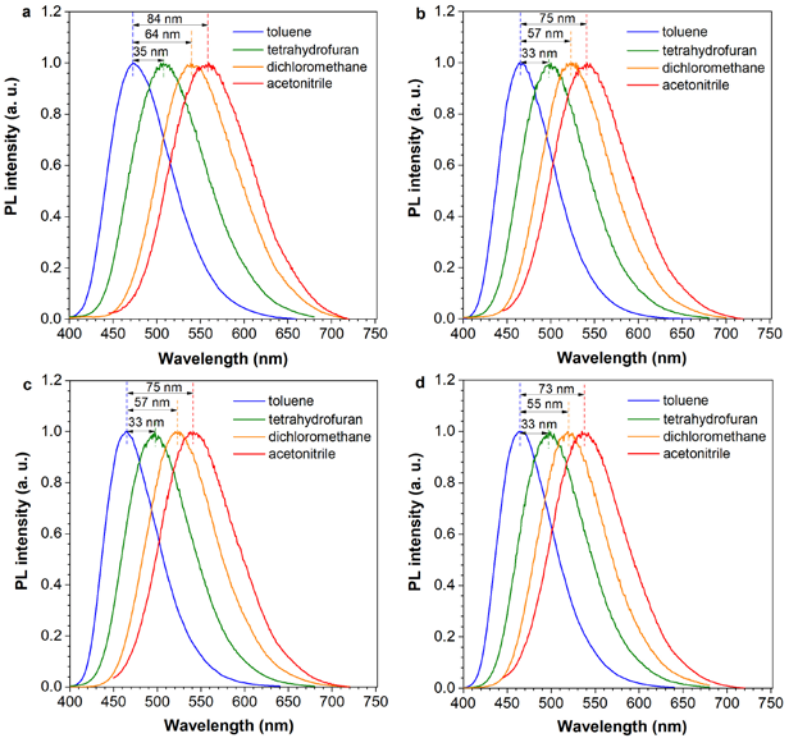

Supplementary Fig. 16 Solvation effect spectra. The PL spectra of (**a**) CBP-1, (**b**) CCO-1, (**c**) CCO-2 and (**d**) CCO-3 in toluene solutions, tetrahydrofuran, dichloromethane and acetonitrile.

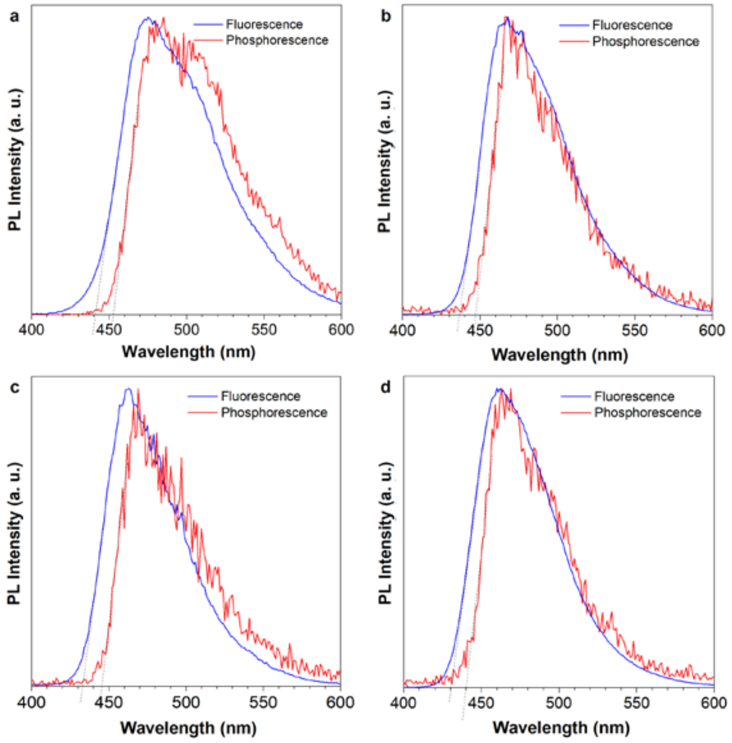

Supplementary Fig. 17 Low temperature fluorescence and phosphorescence spectra. The spectra of (**a**) CBP-1, (**b**) CCO-1, (**c**) CCO-2 and (**d**) CCO-3 in neat films are measured at 77 K under nitrogen.

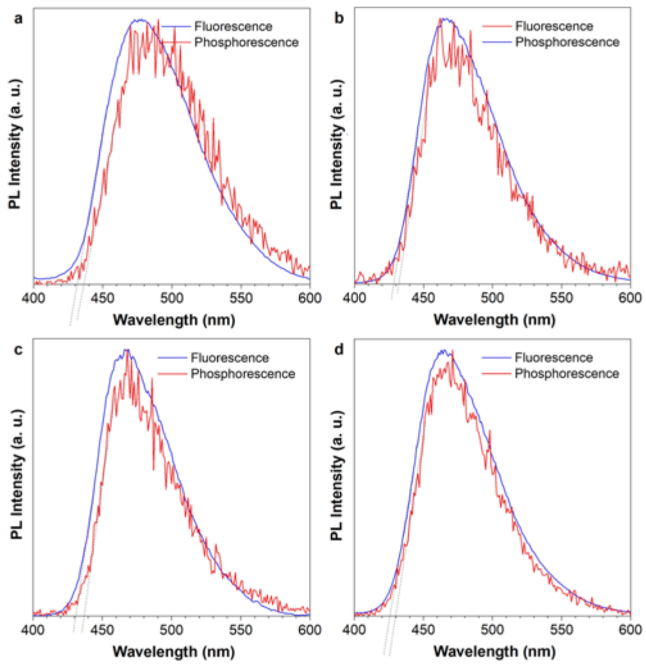

Supplementary Fig. 18 Low temperature fluorescence and phosphorescence spectra. The spectra of (**a**) CBP-1, (**b**) CCO-1, (**c**) CCO-2 and (**d**) CCO-3 in 20 wt% doped films are measured at 77 K under nitrogen.

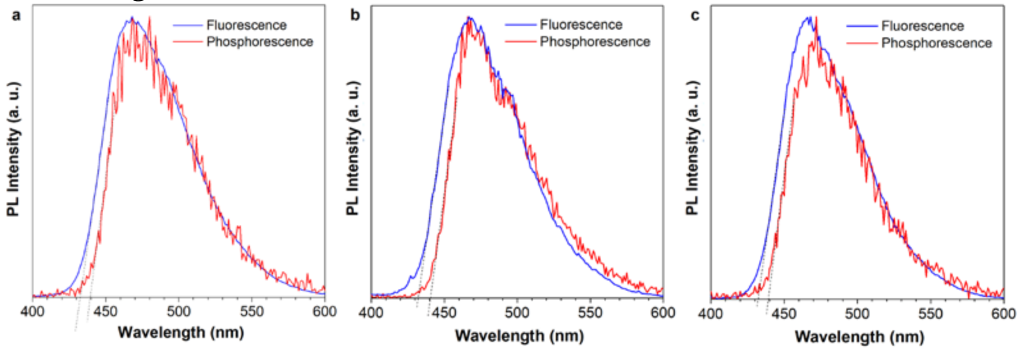

Supplementary Fig. 19 Low temperature fluorescence and phosphorescence spectra. The spectra of (**a**) CCO-1, (**b**) CCO-2 and (**c**) CCO-3 in 40 wt% doped films are measured at 77 K under nitrogen.

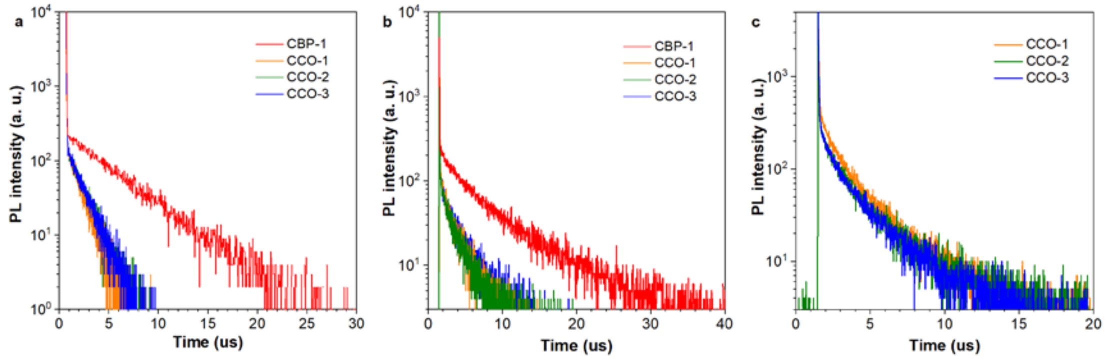

Supplementary Fig. 20 Transient PL decay spectra. The spectra of CBP-1, CCO-1, CCO-2 and CCO-3 in (**a**) toluene and (**b**) neat films, and of CCO-1, CCO-2 and CCO-3 in (**c**) 40 wt% doped films are measured under nitrogen.

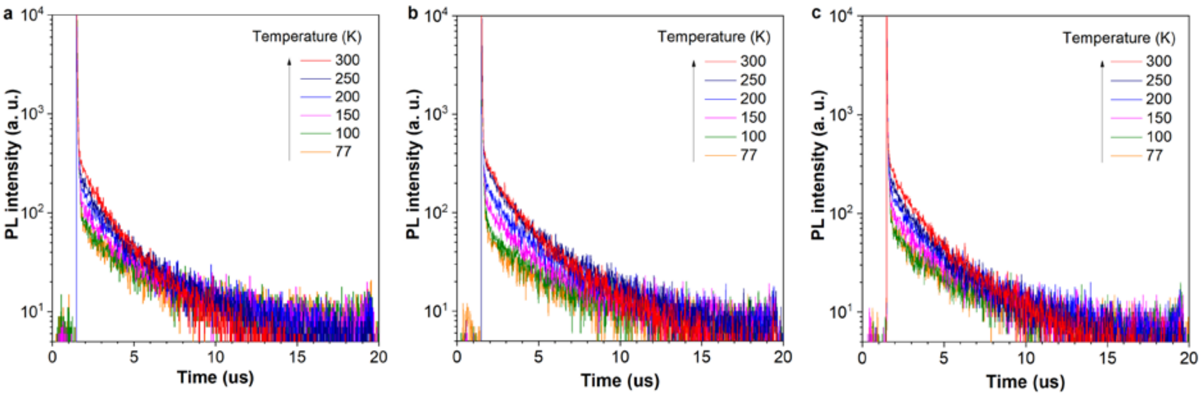

Supplementary Fig. 21 Temperature-dependent transient PL decay spectra. The spectra of (**a**) CCO-1, (**b**) CCO-2 and (**c**) CCO-3 in 20 wt% doped films are measured under nitrogen.

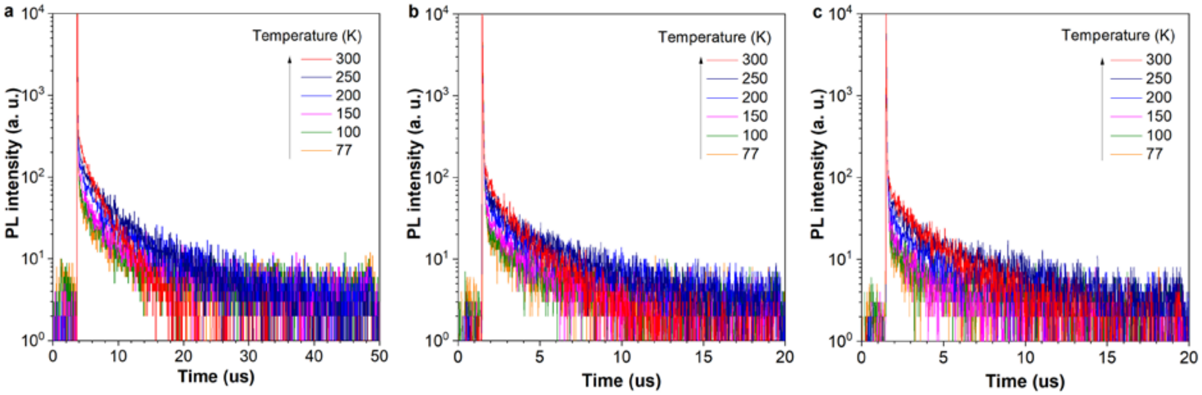

Supplementary Fig. 22 Temperature-dependent transient PL decay spectra. The spectra of (**a**) CCO-1, (**b**) CCO-2 and (**c**) CCO-3 in neat films are measured under nitrogen.

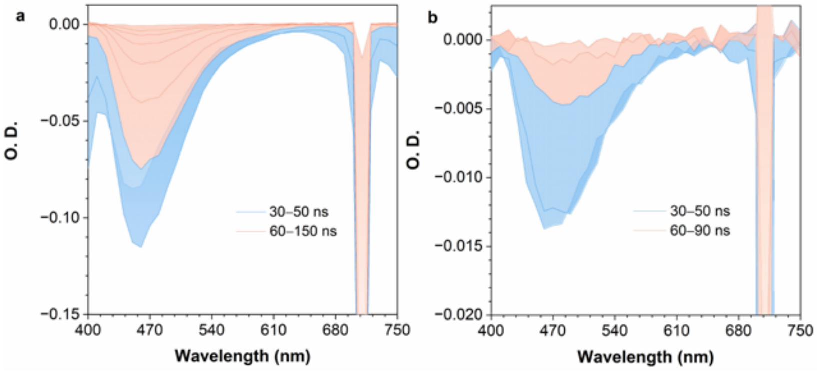

Supplementary Fig. 23 Transient absorption spectra. These spectra of (**a**) CCO-1 and (**b**) CBP-1 on nanosecond timescales are measured in toluene solution (10^{-5} M) under air. Excitation wavelength, 355 nm.

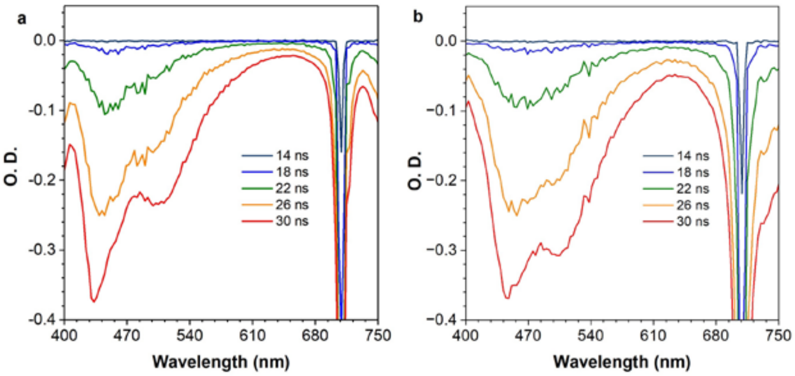

Supplementary Fig. 24 Transient absorption spectra. The spectra of (**a**) CCO-1 and (**b**) CBP-1 at 10–30 ns in toluene solutions (10^{-5} M) under nitrogen atmosphere. Excitation wavelength: 355 nm.

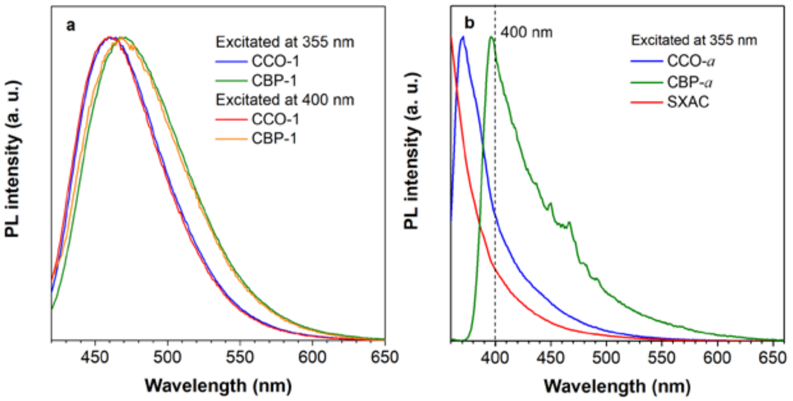

Supplementary Fig. 25 PL spectra in toluene solutions (10^{-5} M) with specific excitation wavelength. PL spectra of (**a**) CCO-1 and CBP-1 with excitation wavelength at 355 and 400 nm, respectively, and of (**b**) CCO-*α*, CBP-*α* and SXAC with excitation wavelength at 355 nm.

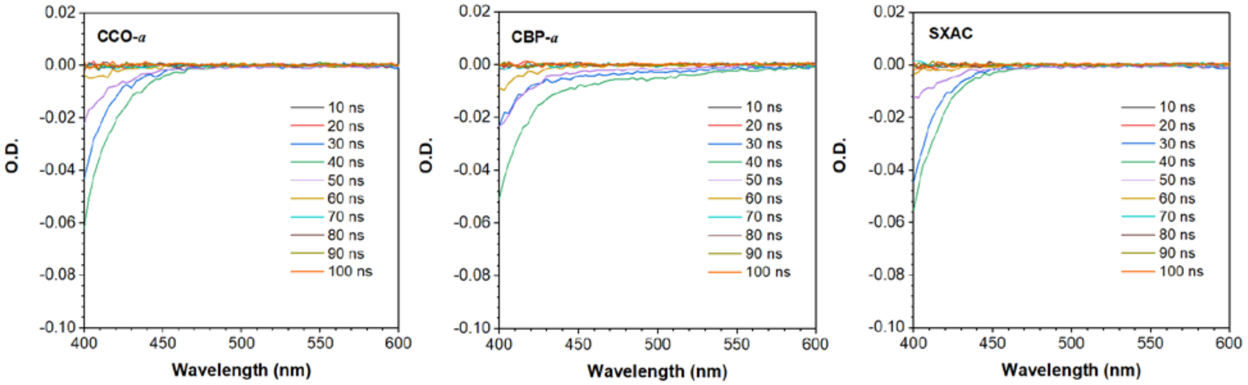

Supplementary Fig. 26 Transient absorption spectra of CCO-*α*, CBP-*α* and SXAC on nanosecond timescales in oxygen-free toluene solutions (10^{-5} M). Excitation wavelength: 355 nm. Discussion and analysis on the transient absorption spectra: From 30 to 100 ns, the prompt fluorescence peak is gradually red-shifted from 432 to 463 nm, while the delayed ones exhibit an opposite trend. The temporal dynamics feature can be ascribed to the various D-A twisting configuration, and thus different CT feature. The initial prompt fluorescence generates by conformers

with weak CT feature, along with fast k_r and large S_1 energy, and then the conformers with relatively strong CT feature emit, leading to the red-shifted emission. In the case of delayed fluorescence, the S_1 configuration with relatively low energy undergo RISC process from T_1 state firstly, owing to their smaller ΔE_{st}, and emit fluorescence, followed by other configurations with relatively high energy, which enable the blue-shift[1]. On the other hand, it is worth noting that before 30 ns, there is only one peak located at ~440 nm (Supplementary Fig. 23). The peak located at 515 nm appears after 30 ns owing to the slower rate of RISC process than that of radiative decay process.

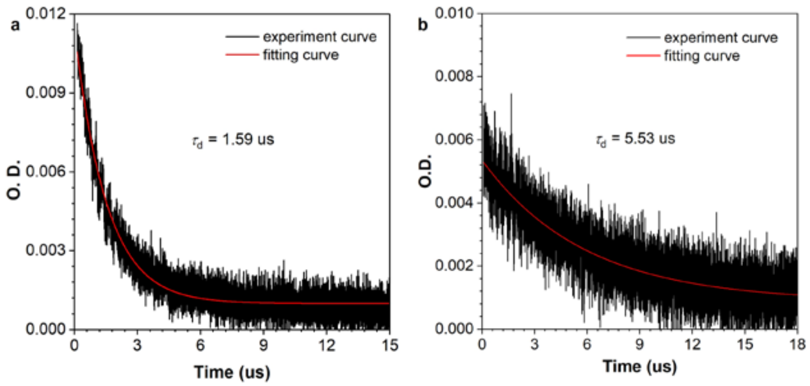

Supplementary Fig. 27 Triplets decay curves. The spectra of (**a**) CCO-1 and (**b**) CBP-1 are measured in toluene solutions (10^{-5} M) under nitrogen atmosphere.

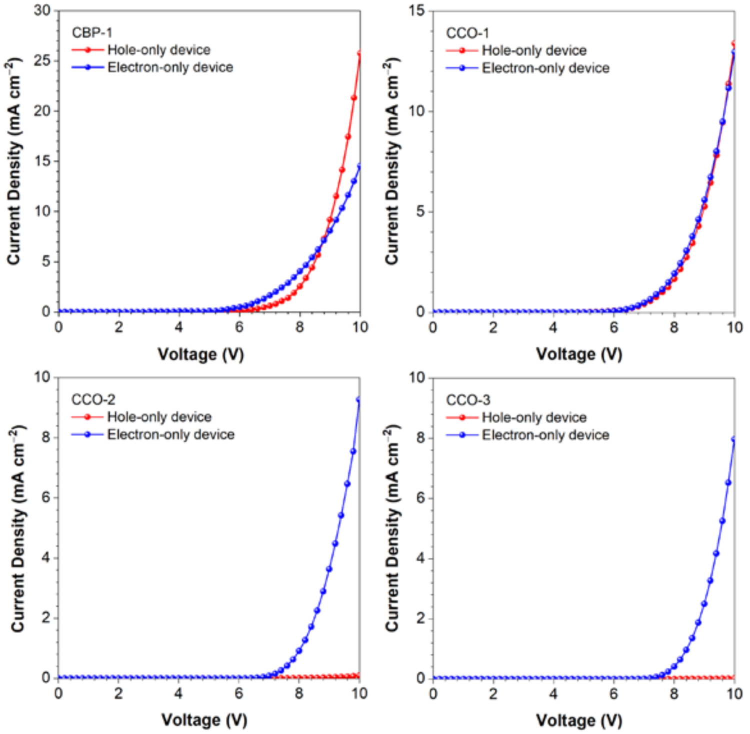

Supplementary Fig. 28 Characterizations of carrier transport ability by space-charge limited current method. These spectra are measured in hole-only and electron-only devices.

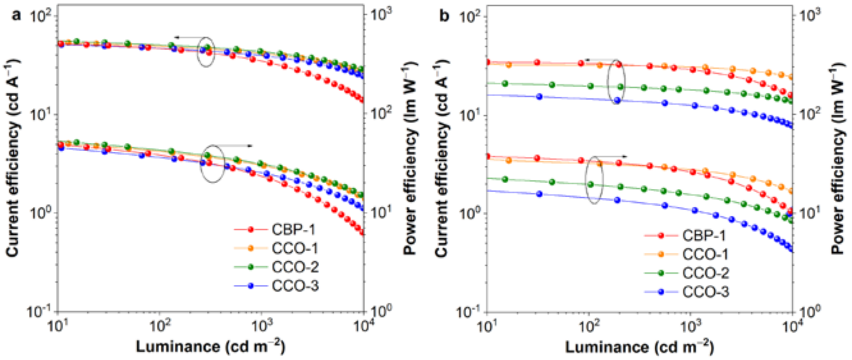

Supplementary Fig. 29 Electroluminescence performance. Current efficiency–luminance–power efficiency curves of (**a**) 20 wt% doped devices and (**b**) nondoped devices of these new molecules.

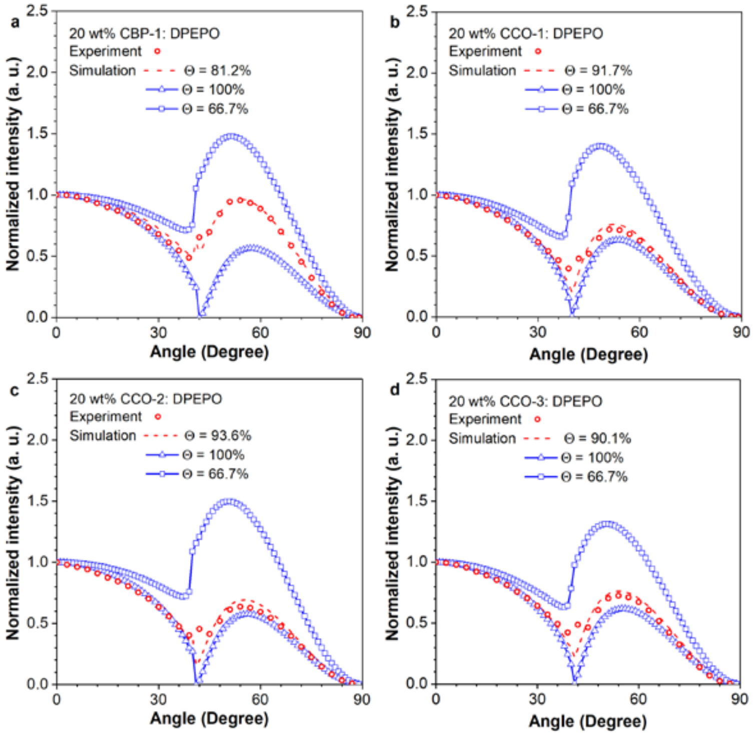

Supplementary Fig. 30 Characterization of horizontal dipole orientation. Measured horizontal transition dipole moment ratios of (**a**) CBP-1, (**b**) CCO-1, (**c**) CCO-2, and (**d**) CCO-3 in 20 wt% doped films.

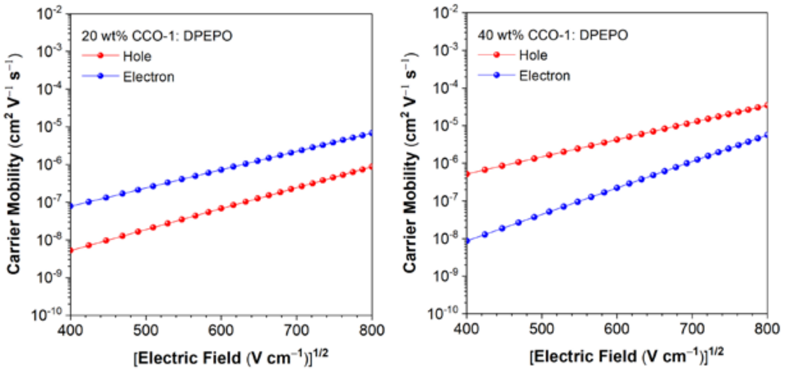

Supplementary Fig. 31 Characterizations of bipolar carrier mobility by SCLC method. Electric field–dependent carrier mobility of 20 and 40 wt% doped films for CCO-2 in single-carrier devices.

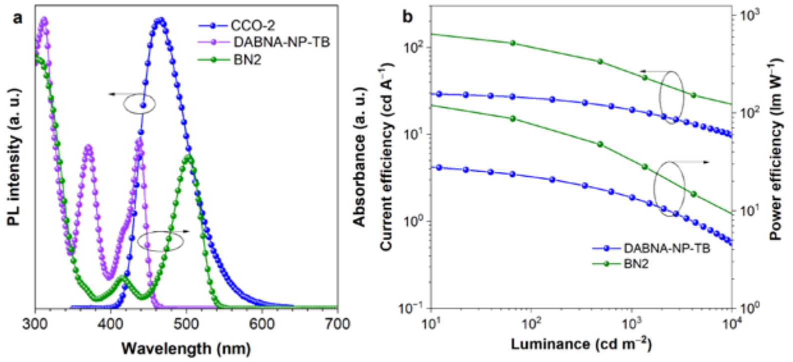

Supplementary Fig. 32 The overlap between absorption and PL spectra and electroluminescence performance. (**a**) Absorption spectra of DABNA-NP-TB, BN2 and PL spectra of CCO-2 in toluene solutions (10^{-5} M). (**b**) Current efficiency–luminance–power efficiency curves of HF OLEDs.

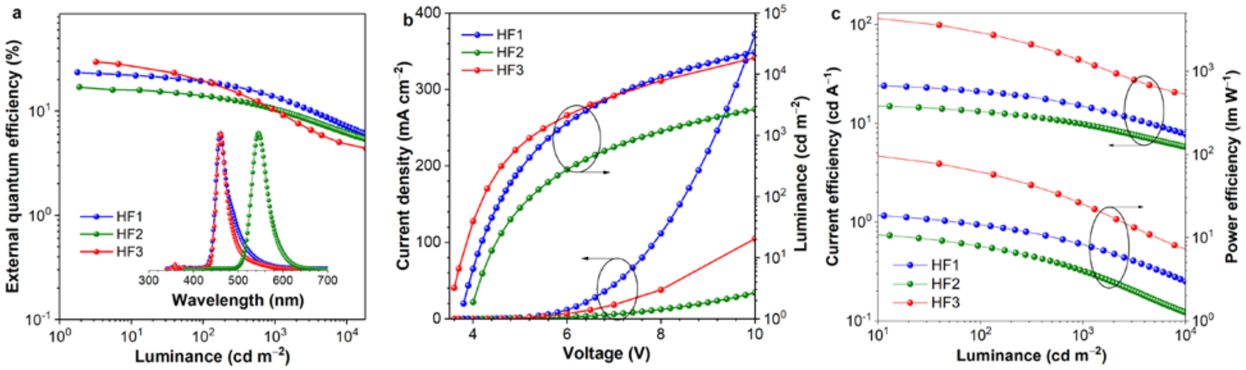

Supplementary Fig. 33 Electroluminescence performance. Plots of (**a**) external quantum efficiency–luminance, (**b**) current density–voltage–luminance and (**c**) current efficiency–luminance–power efficiency spectrum of HF1, HF2 and HF3 devices. Inset in planes (**a**): EL spectra at 4 V.

Supplementary Table 1 Analyses of quantum-chemical calculations results for the new molecules.

	S_1 (eV)[a]	T_1 (eV)[b]	T_2 (eV)[c]	λ_0 (eV)[d]	λ_1 (eV)[e]
CBP-1	2.68	2.51	2.53	0.298	0.253
CCO-1	2.74	2.66	2.78	0.183	0.187
CCO-2	2.75	2.67	2.78	0.184	0.188
CCO-3	2.76	2.68	2.79	0.185	0.193

[a]Energy level of S_1 state. [b]Energy level of T_1 state. [c]Energy level of T_2 state. [d]Reorganization energy of S_0 state. [e]Reorganization energy of S_1 state.

Supplementary Table 2 Calculated radical transition paraments of CBP-1 and CCO-1 in solid state.

	CBP-1	CCO-1
TDM[a]	(–0.027, –0.074, –0.008)	(0.234, 0.066, 0.041)

μ [b]	0.079	0.247
f [c]	0.0004	0.0040
k_r (s^{-1})[d]	1.1×10^5 s^{-1}	1.2×10^6 s^{-1}

[a]Transition dipole moment. [b]Values of transition dipole moment. [c]Oscillator strength. [d]Radiative decay rate.

Supplementary Table 3 Changes on ΔE_{ST} values of neat and doped films, measured at 77 K under nitrogen.

film	F-onset (nm) [a]	S_1 (eV) [b]	P-onset (nm) [c]	T_1 (eV) [d]	ΔE_{ST} (eV)[e]
CBP-1	441	2.812	453	2.737	0.075
20 wt% CBP-1: DPEPO	431	2.877	436	2.844	0.033
CCO-1	437	2.837	447	2.774	0.063
20 wt% CCO-1: DPEPO	428	2.897	433	2.864	0.033
40 wt% CCO-1: DPEPO	432	2.870	439	2.825	0.045
CCO-2	435	2.851	445	2.787	0.064
20 wt% CCO-2: DPEPO	431	2.877	436	2.844	0.033
40 wt% CCO-2: DPEPO	433	2.864	440	2.818	0.046
CCO-3	430	2.884	440	2.818	0.066
20 wt% CCO-3: DPEPO	425	2.918	430	2.884	0.034
40 wt% CCO-3: DPEPO	432	2.870	439	2.825	0.045

[a]Onset of fluorescence spectrum. [b]Energy level of S_1 state. [c]Onset of phosphorescence spectrum. [d]Energy level of T_1 state. [e]Energy gap between S_1 and T_1 states.

Supplementary Table 4 Molecular polarity index (MPI) values of S_0, S_1, T_1 and T_2 states for the new molecules and MPI value of S_0 state for DPEPO.

	MPI_{S0} (kcal/mol)[a]	MPI_{S1} (kcal/mol)[b]	MPI_{T1} (kcal/mol)[c]	MPI_{T2} (kcal/mol)[d]
CBP-1	9.1	16.7	9.6	9.8
CCO-1	8.9	16.5	11.3	8.8
CCO-2	8.8	15.3	10.4	8.6
CCO-3	8.5	14.3	9.7	8.3
DPEPO	11.3	–	–	–

[a]MPI value of S_0 state. [b]MPI value of S_1 state. [c]MPI value of T_1 state. [d]MPI value of T_2 state.

Supplementary Table 5 Photophysical data of the new molecules in toluene solutions.

emitters	τ_{PF} (ns)[a]	τ_{DF} (μs)[b]	Φ_{PL} (%)[c]	k_r ($\times 10^7$ s^{-1})[d]	k_{RISC} ($\times 10^5$ s^{-1})[e]
CBP-1	27	4.35	25	0.67	3.54
CCO-1	25	1.64	70	1.63	9.43
CCO-2	25	1.54	72	1.73	10.67
CCO-3	23	1.54	69	1.67	10.68

[a]Lifetime of prompt fluorescence. [b]Lifetime of delayed fluorescence. [c]Absolute photoluminescence quantum yield. [d]Radiative decay rate. [e]Reverse intersystem crossing rate. The transition rates are calculated according to the method described in Ref. 2.

Supplementary Table 6 Photophysical data of neat and doped films of the new molecules.

emitter	τ_{PF} (ns)[a]	τ_{DF} (μs)[b]	Φ_{PL} (%)[c]	k_r ($\times 10^7$ s^{-1})[d]	k_{RISC} ($\times 10^5$ s^{-1})[e]	ΔE_{ST} (meV)[f]
20 wt% doped films						
CBP-1	25	7.55	85	1.32	3.43	33
CCO-1	24	1.91	99	1.54	13.81	33

CCO-2	24	1.80	99	1.66	13.74	33
CCO-3	23	1.91	99	1.58	14.16	34
40 wt% doped films						
CCO-1	23	1.87	95	1.86	11.7	45
CCO-2	23	1.89	97	1.87	11.6	46
CCO-3	22	1.91	95	1.87	11.9	45
neat films						
CBP-1	21	5.41	63	1.58	3.72	75
CCO-1	22	1.69	68	2.34	7.68	63
CCO-2	20	1.61	72	2.61	8.12	64
CCO-3	19	1.63	66	2.73	7.73	66

[a]Lifetimes of prompt fluorescence. [b]Lifetime of delayed fluorescence. [c]Absolute photoluminescence quantum yield. [d]Radiative decay rate. [e]Reverse intersystem crossing rate. [f]Energy gap between S_1 and T_1 states.

Supplementary Table 7 EL performances of the new molecules in doped devices and representative blue and deep-blue TADF emitters with $CIE_y < 0.2$ in literatures.

emitting layer	λ_{EL} (nm)[a]	$CIE_{x,y}$[b]	$\eta_{ext,max}$ (%)[c]	$\eta_{ext,1000}$ (%)[d]	Ref.
20 wt% CCO-2: DPEPO	470	(0.14, 0.18)	43.4	31.3	this work
20 wt% CCO-1: DPEPO	470	(0.14, 0.19)	41.8	29.4	this work
20 wt% CCO-3: DPEPO	466	(0.14, 0.17)	40.5	30.0	this work
10 wt% CCO-2: DPEPO	462	(0.14, 0.15)	41.3	22.4	this work
10 wt% CCO-3: DPEPO	462	(0.14, 0.14)	37.7	20.2	this work
20 wt% 2TBCz-XT: PPF	462	(0.15, 0.18)	33.7	–	4
50 wt% OBO-II: PPF	468	(0.14, 0.17)	33.8	26.2	5
20 wt% TDBA-DI: PPBI	–	(0.14, 0.15)	32.23	26.75	3
7 wt% 3DPyM-*p*DTC: *m*CBP	464	(0.14, 0.18)	31.9	–	6
20 wt% OBO-II: PPF	464	(0.14, 0.13)	31.7	20.8	5
10 wt% OBOtSAc: DPEPO	452	(0.147, 0.092)	31.2	–	7
70 wt% OBO-II: PPF	470	(0.14, 0.19)	30.4	23.4	5
10 wt% tBuOBOtSAc: DPEPO	448	(0.149, 0.061)	28.2	–	7
20 wt% TDBA-SAF: DPEPO	456	(0.142, 0.090)	28.1	17.6	9
20 wt% 2PhCz2CzBN: *m*CBP	464	(0.154, 0.200)	26.6	11.7	10
20 wt% TDBA-Ac: DBFPO	–	(0.14, 0.15)	25.71	18.92	3
30 wt% sAC-sDBB: *m*CP	444	(0.151, 0.058)	25.4	20.0	11
20 wt% MCz-XT: PPF	460	(0.15, 0.15)	24.0	–	4
30 wt% 2tCz2CzBN: *m*CBP	464	(0.153, 0.193)	23.8	12.4	10
15 wt% p4TCzPhBN: DPEPO	–	(0.15, 0.19)	22.8	20.4	13
10 wt% Cz-XT: DPEPO	442	(0.15, 0.08)	22.2	–	4
20 wt% PX-SBA: DPEPO	448	(0.16, 0.15)	20.8	7	8
30 wt% TMCz-BO: PPF	471	(0.14, 0.18)	20.7	17.4	14
20 wt% QBO: PPF	460	(0.14, 0.12)	20.5	17.7	12

[a]EL peak wavelength. [b]Commission Internationale de I'Eclairage coordinates. [c]Maximum external quantum efficiency. [d]External quantum efficiency at 1000 cd m^{-2}.

Supplementary Table 8 EL performances of CBP-1 in nondoped and doped OLEDs.

CBP-1	V_{on} (V)[a]	$\eta_{C,max}$ (cd A^{-1})[b]	$\eta_{P,max}$ (lm W^{-1})[c]	$\eta_{ext,max}$ (%)[d]	$\eta_{ext,1000}$ (%)[e]	Roll-off (%)[f]	L_{max} (cd m^{-2})[g]	$CIE_{x,y}$[h]	λ_{EL} (nm)[i]
100 wt%	2.8	36.0	40.3	20.5	16.5	19.4	23880	(0.16, 0.25)	474
10 wt%	3.7	42.1	35.7	30.2	13.0	56.9	4741	(0.15, 0.18)	468
20 wt%	3.2	54.0	53.0	33.8	22.0	34.9	16640	(0.15, 0.22)	474

30 wt%	2.9	54.4	53.4	30.9	23.9	22.5	29970	(0.16, 0.25)	476

[a]Turn-on voltage at 1 cd m^{-2}. [b]Maximum current efficiency. [c]Maximum power efficiency. [d] Maximum external quantum efficiency. [e]External quantum efficiency at 1000 cd m^{-2}. [f]External quantum efficiency roll-off at 1000 cd m^{-2}. [g]Maximum luminance. [h]Commission Internationale de I'Eclairage coordinates. [i]EL peak wavelength.

Supplementary Table 9 EL performances of devices HF1, HF2 and HF3.

Device	V_{on} (V)[a]	$\eta_{C,max}$ (cd A^{-1})[b]	$\eta_{P,max}$ (lm W^{-1})[c]	$\eta_{ext,max}$ (%)[d]	L_{max} (cd m^{-2})[f]	$CIE_{x,y}$[g]	λ_{EL} (nm)[h]
HF1	3.8	25.6	21.2	23.5	45130	(0.14, 0.12)	460
HF2	4.0	15.9	12.5	17.0	30440	(0.14, 0.10)	460
HF3	3.6	124.0	108.2	29.7	40590	(0.33, 0.63)	544

[a]Turn-on voltage at 1 cd m^{-2}. [b]Maximum current efficiency. [c]Maximum power efficiency. [d]Maximum external quantum efficiency. [e]External quantum efficiency at 1000 cd m^{-2}. [f]Maximum luminance. [g]Commission Internationale de I'Eclairage coordinates. [h]EL peak wavelength.

Supplementary Table 10 EL performances of the HF OLEDs with CCO-2 as sensitizer and MR-TADF molecules as emitters, and representative blue (CIE_y < 0.15) and green (CIE_y > 0.60) bottom-emitting vacuum evaporated OLEDs based on MR-TADF emitters.

Emitting layer	λ_{EL} (nm)[a]	$CIE_{x,y}$[b]	$\eta_{ext,max}$ (%)[c]	$L_{initial}$ (cd m^{-2})[d]	LT_x (h)[e]	Ref.
Blue MR-TADF molecule						
1 wt% DABNA-NP-TB: 20 wt% CCO-2: 2,6-DCzPPy (HF1)	460	(0.14, 0.12)	23.5	1000	9.8 (LT_{50})	this work
1 wt% DABNA-NP-TB: 20 wt% CCO-2: 2,6-DCzPPy (HF2)	460	(0.14, 0.10)	17.0	1000	21.9 (LT_{50})	this work
1 wt% DABNA-NP-TB: DOBNA-Tol	457	(0.14, 0.11)	19.5	100	19 (LT_{80})	15
3 wt% BOBO-Z: mCBP-CN	445	(0.16, 0.06)	2.3	100	4.4 (LT_{50})	16
3 wt% BOBS-Z: mCBP-CN	455	(0.14, 0.06)	24.2	100	34.0 (LT_{50})	16
3 wt% BSBS-Z: mCBP-CN	463	(0.13, 0.08)	24.0	100	37.4 (LT_{50})	16
1wt% ν-DABNA: DOBNA-OAr	469	(0.12, 0.11)	34.4	100	31 (LT_{50})	17
1 wt% *v*-DABNA-O-Me: DOBNA-Tol	465	(0.13, 0.10)	29.5	100	314 (LT_{50})	18
4 wt% C-BN: 20 wt% p4TzPhBN: mCBP	453	(0.14, 0.07)	26.6	500	12.3 (LT_{80})	19
1 wt% pSFIAc1: 30 wt% m4TCzPhBN: mCBP	446	(0.148, 0.058)	24.9	1000	13 (LT_{50})	20
1 wt% pSFIAc2: 30 wt% m4TCzPhBN: mCBP	451	(0.146, 0.078)	31.4	1000	20 (LT_{50})	20
2wt% *t*-DABNA: 40 wt% p4TCzPhBN: mCPCz	–	(0.13, 0.12)	32.5	1000	60 (LT_{80})	21
1 wt%: *v*-DABNA: oCBP:CNmCBPCN	–	(0.13, 0.14)	33.2	1000	41 (LT_{50})	22
Green MR-TADF molecule						
3 wt% BN2: 20 wt%CCO-2: 2,6-DCzPPy	544	(0.33, 0.63)	29.7	1000	691.7 (LT_{50})	this work
1 wt% CzBO: mCBP	448	(0.15, 0.05)	13.4	100	0.16 (LT_{50})	23
1 wt% CzBS: mCBP	473	(0.11, 0.16)	23.1	100	4.23 (LT_{50})	23
1 wt% CzBSe: mCBP	481	(0.10, 0.24)	23.9	100	7.48 (LT_{50})	23
1 wt% TCz-B: mCBP	515	(0.16, 0.71)	29.2	100	2.0 (LT_{50})	24
1 wt% γ-Cb-B: oCBP	461	(0.13, 0.13)	19.0	100	0.9 (LT_{50})	24

1 wt% *m*-CzB: mCBP-CN	515	(0.20, 0.70)	23.5	100	25.5 (LT_{50})	25
1 wt% *p*-CzB: mCBP-CN	511	(0.16, 0.66)	20.2	100	6.4 (LT_{50})	25
1wt% DBNO: PhCzBCz	504	(0.18, 0.60)	35.9	500	0.29 (LT_{50})	26
:1 wt% BN-DPAC: mCBP:PO-T2T (1:1)	508	(0.16, 0.61)	30.2	500	8 (LT_{50})	27
0.5 wt% *v*-DABNA-CN-Me: DOBNA-Ph	504	(0.13, 0.65)	31.9	800	59 (LT_{80})	28
1 wt% 2PXZBN: DMIC-TRZ	517	(0.23, 0.67)	30.7	1000	158 (LT_{50})	29
1 wt% 2PXTBN: DMIC-TRZ	520	(0.24, 0.67)	34.6	1000	5.6 (LT_{50})	29
1 wt% BNSSe: DMIC-TRZ	515	(0.22, 0.66)	35.7	1000	4.8(LT_{50})	29
1 wt% BNSeSe: DMIC-TRZ	512	(0.19, 0.66)	36.8	1000	4.1 (LT_{50})	29
1 wt % BN-Se: DMIC-TRZ	506	(0.15, 0.62)	32.6	1000	82.1 (LT_{50})	30
1 wt % DCzBN-Au: DMIC-TRZ	510	(0.16, 0.67)	35.8	1000	150.1(LT_{80})	31
9 wt% 2F-BN: 35 wt% 5TCzBN: mCPCB	501	(0.16, 0.60)	22.0	2000	45.76(LT_{90})	32
6 wt% AZA-BN: 30 wt%Ir(ppy)$_3$: mCBP	527	(0.27, 0.69)	28.2	2000	46.3 (LT_{90})	33
3wt% BN-ICz-1: 30wt% 3CTF: mCBP	523	(0.22, 0.74)	30.5	2000	82.3 (LT_{90})	34
3wt% BN-ICz-2: 30wt% 3CTF: mCBP	523	(0.21, 0.73)	29.8	2000	71.3 (LT_{90})	34
2 wt% tCzphB-Ph: 5wt% Ir(ppy)$_3$: BCz-*o*-TRZ (structure I)	526	(0.21, 0.74)	31.3	2.33×10^4	0.5 (LT_{90})	35
2 wt% tCzphB-FI: 5wt% Ir(ppy)$_3$: BCz-*o*-TRZ (structure I)	535	(0.26, 0.71)	29.7	2.45×10^4	12.0 (LT_{90})	35
2 wt% tCzphB-Ph: 5wt% Ir(ppy)$_3$: BCz-*o*-TRZ (structure II)		(0.20, 0.73)	~31.3	2.14×10^4	1.4 (LT_{90})	35
2 wt% tCzphB-FI: 5wt% Ir(ppy)$_3$: BCz-*o*-TRZ (structure II)		(0.26, 0.71)	~29.7	2.40×10^4	70.5 (LT_{90})	35

[a]EL peak wavelength. [b]Commission Internationale de I'Eclairage coordinates. [c]Maximum external quantum efficiency. [d]Initial luminance for testing device stability. [e]Times of luminance decay to *x*% of the initial luminance.

Configurations of OLEDs

1) The configuration of nondoped OLEDs

ITO/HATCN (5 nm)/TAPC (50 nm)/TcTa (5 nm)/*m*CP (5 nm)/CBP-1 (20 nm)/DPEPO (5 nm)/TmPyPB (30 nm)/LiF (1 nm)/Al

ITO/HATCN (5 nm)/TAPC (50 nm)/TcTa (5 nm)/*m*CP (5 nm)/CCO-1 (20 nm)/DPEPO (5 nm)/TmPyPB (30 nm)/LiF (1 nm)/Al

ITO/HATCN (5 nm)/TAPC (50 nm)/TcTa (5 nm)/*m*CP (5 nm)/CCO-2 (20 nm)/DPEPO (5 nm)/TmPyPB (30 nm)/LiF (1 nm)/Al

ITO/HATCN (5 nm)/TAPC (50 nm)/TCTA (5 nm)/*m*CP (5 nm)/CCO-3 (20 nm)/DPEPO (5 nm)/TmPyPB (30 nm)/LiF (1 nm)/Al

2) The configuration of doped OLEDs

ITO/HATCN (5 nm)/TAPC (50 nm)/TcTa (5 nm)/*m*CP (5 nm)/*x* wt% CBP-1: DPEPO (20 nm)/DPEPO (5 nm)/TmPyPB (30 nm)/LiF (1 nm)/Al (x = 10, 20, 30)

ITO/HATCN (5 nm)/TAPC (50 nm)/TcTa (5 nm)/*m*CP (5 nm)/*x* wt% CCO-1: DPEPO (20 nm)/DPEPO (5 nm)/TmPyPB (30 nm)/LiF (1 nm)/Al (x = 10, 20, 30, 40 and 50)

ITO/HATCN (5 nm)/TAPC (50 nm)/TcTa (5 nm)/mCP (5 nm)/*x* wt% CCO-2: DPEPO (20 nm)/DPEPO (5 nm)/TmPyPB (30 nm)/LiF (1 nm)/Al (*x* = 10, 20, 30, 40 and 50)
ITO/HATCN (5 nm)/TAPC (50 nm)/TcTa (5 nm)/mCP (5 nm)/*x* wt% CCO-3: DPEPO (20 nm)/DPEPO (5 nm)/TmPyPB (30 nm)/LiF (1 nm)/Al (*x* = 10, 20, 30, 40 and 50)
3) The configuration of the HF devices
ITO/HATCN (5 nm)/TAPC (50 nm)/TCTA (5 nm)/*m*CP (5 nm)/1 wt% DABNA-NP-TB: 40 wt% CCO-2: *m*CPBC (20 nm)/DPEPO (5 nm)/TmPyPB (30 nm)/LiF (1 nm)/Al
ITO/HATCN (5 nm)/TAPC (50 nm)/TCTA (5 nm)/*m*CP (5 nm)/1 wt% BN2: 40 wt% CCO-2: 2,6-DCzPPy (20 nm)/DPEPO (5 nm)/TmPyPB (30 nm)/LiF (1 nm)/Al
4) The configuration of HF1, HF2 and HF3
HF1: ITO/MoO_3 (6 nm)/*m*CBP (40 nm)/1 wt% DABNA-NP-TB: 20 wt% CCO-2: 2,6-DCzPPy (30 nm)/DBFTRZ (5 nm)/Bpy-TP2 (30 nm)/LiF (2 nm)/Al
HF2: ITO/MoO_3 (6 nm)/CzSi (4 nm)/NPB (350 nm)/BCzPh (10 nm)/1 wt% DABNA-NP-TB: 20 wt% CCO-2: 2, 6-DCzPPy (30 nm)/46DCzPPM (10 nm)/DPPyA (30 nm)/LiF (1 nm)/Al
HF3: ITO/MoO_3 (6 nm)/CzSi (4 nm)/NPB (180 nm)/BCzPh (10 nm)/3 wt% BN2: 20 wt% CCO-2: 2, 6-DCzPPy (30 nm)/46DCzPPM (10 nm)/DPPyA (30 nm)/LiF (1 nm)/Al
5) The configurations of hole-only devices:
ITO/TAPC (50 nm)/CCO-1 (20 nm)/TAPC (40 nm)/Al
ITO/TAPC (50 nm)/CCO-2 (20 nm)/TAPC (40 nm)/Al
ITO/TAPC (50 nm)/CCO-3 (20 nm)/TAPC (40 nm)/Al
ITO/TAPC (50 nm)/CBP-1 (20 nm)/TAPC (40 nm)/Al
ITO/TAPC (50 nm)/20 wt% CCO-2: DPEPO (20 nm)/TAPC (40 nm)/Al
ITO/TAPC (50 nm)/40 wt% CCO-2: DPEPO (20 nm)/TAPC (40 nm)/Al
6) The configurations of electron-only devices:
ITO/TmPyPB (50 nm)/CCO-1 (20 nm)/TmPyPB (40 nm)/LiF (1 nm)/Al
ITO/TmPyPB (50 nm)/CCO-2 (20 nm)/TmPyPB (40 nm)/LiF (1 nm)/Al
ITO/TmPyPB (50 nm)/CCO-3 (20 nm)/TmPyPB (40 nm)/LiF (1 nm)/Al
ITO/TmPyPB (50 nm)/CBP-1 (20 nm)/TmPyPB (40 nm)/LiF (1 nm)/Al
ITO/TmPyPB (50 nm)/20 wt% CCO-2: DPEPO (20 nm)/TmPyPB (40 nm)/LiF (1 nm)/Al
ITO/TmPyPB (50 nm)/40 wt% CCO-2: DPEPO (20 nm)/TmPyPB (40 nm)/LiF (1 nm)/Al

References

1. Serevicius, T., et al. Temporal Dynamics of Solid-State Thermally Activated Delayed Fluorescence: Disorder or Ultraslow Solvation? *J. Phys. Chem. Lett.* **13**, 1839–1844 (2022).
2. Zhang, Q., et al. Efficient blue organic light-emitting diodes employing thermally activated delayed fluorescence. *Nat. Photon.* **8**, 326–332 (2014).
3. Ahn, D.H., et al. Highly efficient blue thermally activated delayed fluorescence emitters based on symmetrical and rigid oxygen-bridged boron acceptors. *Nat. Photon.* **13**, 540–546 (2019).
4. Chen, J., et al. Towards efficient blue delayed-fluorescence molecules by modulating torsion angle between electron donor and acceptor. *CCS Chem.* **5**, 598–606 (2023).
5. Park, I.S., Min, H., Kim, J.U. & Yasuda, T. Deep-blue OLEDs based on organoboron–phenazasiline-hybrid delayed fluorescence emitters concurrently achieving 30% external quantum efficiency and small efficiency roll-off. *Adv. Opt. Mater.* **9**, 2101282 (2021).
6. Rajamalli, P., et al. New molecular design concurrently providing superior pure blue, thermally activated delayed fluorescence and optical out-coupling efficiencies. *J. Am. Chem. Soc.* **139**,

10948–10951 (2017).
7. Lee, Y. & Hong, J.-I. High-efficiency thermally activated delayed fluorescence emitters with high horizontal orientation and narrow deep-blue emission. *Adv. Opt. Mater.* **9**, 2100406 (2021).
8. Liu, M., et al. Horizontally orientated sticklike emitters: enhancement of intrinsic out-coupling factor and electroluminescence performance. *Chem. Mater.* **29**, 8630–8636 (2017).
9. Lim, H., et al. Highly efficient deep-blue OLEDs using a TADF emitter with a narrow emission spectrum and high horizontal emitting dipole ratio. *Adv. Mater.* **32**, 2004083 (2020).
10. Zou, S.-J., et al. High-performance nondoped blue delayed fluorescence organic light-emitting diodes featuring low driving voltage and high brightness. *Adv. Sci.* **7**, 1902508 (2020).
11. Xia, G., et al. A TADF emitter featuring linearly arranged spiro-donor and spiro-acceptor groups: efficient nondoped and doped deep-blue OLEDs with CIEy <0.1. *Angew. Chem. Int. Ed.* **60**, 9598–9603 (2021).
12. Min, H., Park, I.S., Yasuda, T. Blue Thermally activated delayed fluorescence with sub-microsecond short exciton lifetimes: acceleration of triplet–singlet spin interconversion via quadrupolar charge-transfer states. *Adv. Opt. Mater.* **10**, 2200290 (2022).
13. Zhang, D., et al. Efficient and stable deep-blue fluorescent organic light-emitting diodes employing a sensitizer with fast triplet upconversion. *Adv. Mater.* **32**, 1908355 (2020).
14. Kim, J.U. et al. Nanosecond-time-scale delayed fluorescence molecule for deep-blue OLEDs with small efficiency rolloff. *Nat. Commun.* **11**, 1765 (2020).
15. Oda, S., et al. Carbazole-Based DABNA Analogues as Highly Efficient Thermally Activated Delayed Fluorescence Materials for Narrowband Organic Light-Emitting Diodes. *Angew. Chem. Int. Ed.* **60**, 2882–2886 (2021).
16. Park, I.S., Yang, M., Shibata, H., Amanokura, N. & Yasuda, T. Achieving Ultimate Narrowband and Ultrapure Blue Organic Light-Emitting Diodes Based on Polycyclo-Heteraborin Multi-Resonance Delayed-Fluorescence Emitters. *Adv. Mater.* **34**, e2107951 (2022).
17. Kondo, Y., et al. Narrowband deep-blue organic light-emitting diode featuring an organoboron-based emitter. *Nat. Photon.* **13**, 678–682 (2019).
18. Tanaka, H., et al. Hypsochromic Shift of Multiple-Resonance-Induced Thermally Activated Delayed Fluorescence by Oxygen Atom Incorporation. *Angew. Chem. Int. Ed.* **60**, 17910–17914 (2021).
19. Fan, T., et al. One-Shot Synthesis of B/N-Doped Calix[4]arene Exhibiting Narrowband Multiple Resonance Fluorescence. *Angew. Chem. Int. Ed.* **61**, e202213585 (2022).
20. Meng, G., et al. Highly efficient and stable deep-blue OLEDs based on narrowband emitters featuring an orthogonal spiro-configured indolo[3,2,1-de]acridine structure. *Chem. Sci.* **13**, 5622–5630 (2022).
21. Zhang, D., et al. Efficient and Stable Deep-Blue Fluorescent Organic Light-Emitting Diodes Employing a Sensitizer with Fast Triplet Upconversion. *Adv. Mater.* **32**, e1908355 (2020).
22. Jeon, S.O., et al. High-efficiency, long-lifetime deep-blue organic light-emitting diodes. *Nat. Photon.* **15**, 208–215 (2021).
23. Park, I.S., Min, H. & Yasuda, T. Ultrafast Triplet-Singlet Exciton Interconversion in Narrowband Blue Organoboron Emitters Doped with Heavy Chalcogens. *Angew. Chem. Int. Ed.* **61**, e202205684 (2022).
24. Yang, M., et al. Wide-Range Color Tuning of Narrowband Emission in Multi-resonance Organoboron Delayed Fluorescence Materials through Rational Imine/Amine Functionalization. *Angew. Chem. Int. Ed.* **60**, 23142–23147 (2021).
25. Yang, M., Konidena, R.K., Shikita, S. & Yasuda, T. Facile dimerization strategy for producing

narrowband green multi-resonance delayed fluorescence emitters. *J. Mater. Chem. C* **11**, 917–922 (2022).
26. Cai, X., et al. Achieving 37.1% Green Electroluminescent Efficiency and 0.09 eV Full Width at Half Maximum Based on a Ternary Boron-Oxygen-Nitrogen Embedded Polycyclic Aromatic System. *Angew. Chem. Int. Ed.* **61**, e202200337 (2022).
27. Jiang, P., et al. Simple Acridan-Based Multi-Resonance Structures Enable Highly Efficient Narrowband Green TADF Electroluminescence. *Adv. Opt. Mater.* **9**, 2100825 (2021).
28. Oda, S., et al. Development of Pure Green Thermally Activated Delayed Fluorescence Material by Cyano Substitution. *Adv. Mater.* **34**, 2201778 (2022).
29. Hu, Y. X. et al, Efficient Selenium-Integrated TADF OLEDs with Reduced Roll-Off. *Nat. Photon.* **16**, 803–810 (2022).
30. Cao, X., et al. Manipulating Exciton Dynamics toward Simultaneous High-Efficiency Narrow-band Electroluminescence and Photon Upconversion by a Selenium-Incorporated Multiresonance Delayed Fluorescence Emitter. *J. Am. Chem. Soc.* **144**, 22976−22984 (2022).
31. Wang, J., et al. Metal-Perturbed Multi-Resonance TADF Emitter Enables High-efficiency and Ultralow Efficiency Roll-off Non-Sensitized OLEDs with Pure Green Gamut. *Adv. Mater.* **35**, 2208378 (2023).
32. Zhang, Y., et al. Multi-Resonance Induced Thermally Activated Delayed Fluorophores for Narrowband Green OLEDs. *Angew. Chem. Int. Ed.* **58**, 16912–16917 (2019).
33. Zhang, Y., et al, Achieving Pure Green Electroluminescence with CIEy of 0.69 and EQE of 28.2% from an Aza-Fused Multi-Resonance Emitter. *Angew. Chem. Int. Ed*. **59**, 17499–17503 (2020).
34. Zhang, Y., et al. Fusion of Multi-Resonance Fragment with Conventional Polycyclic Aromatic Hydrocarbon for Nearly BT.2020 Green Emission. *Angew. Chem. Int. Ed.* **61**, e202202380 (2022).
35. Liu, J., et al, Toward a BT.2020 Green Emitter Through a Combined Multiple Resonance Effect and Multi-Lock Strategy. *Nat. Commun.* **13**, 4876 (2022).
