# Supplementary material for: Realizing efficient blue and deep-blue delayed fluorescence materials with record-beating electroluminescence efficiencies of 43.4%
Source: Nat Commun. 2023 Apr 10;14:2019. doi: 10.1038/s41467-023-37687-3 (PMC10086064; doi:10.1038/s41467-023-37687-3)
Supplement: Supplementary file 3 — Description of Additional Supplementary Files [file 41467_2023_37687_MOESM3_ESM.pdf]

Description of Additional Supplementary Files

File Name: Supplementary Data 1
Description: Crystal structure of CBP-1 (CCDC: 2209209) cultured in dichloromethane/n-hexane.

File Name: Supplementary Data 2
Description: Crystal structure of CCO-1 (CCDC: 2209210) cultured in chloroform/*n*-hexane.

File Name: Supplementary Data 3
Description: Crystal structure of CCO-2 (CCDC: 2209211) cultured in deuterated chloroform.

File Name: Supplementary Data 4
Description: Crystal structure of CCO-3 (CCDC: 2209436) cultured in chloroform/ethanol.
